# Crystal structure and Hirshfeld surface analysis of 2-{[2,8-bis­(tri­fluoro­meth­yl)quinolin-4-yl](hy­droxy)meth­yl}piperidin-1-ium 2-hy­droxy-2-phenyl­acetate hemihydrate

**DOI:** 10.1107/S2056989016016492

**Published:** 2016-10-25

**Authors:** James L. Wardell, Mukesh M. Jotani, Edward R. T. Tiekink

**Affiliations:** a Fundaçaö Oswaldo Cruz, Instituto de Tecnologia em Fármacos-Far Manguinhos, 21041-250 Rio de Janeiro, RJ, Brazil; b Department of Chemistry, University of Aberdeen, Old Aberdeen, AB24 3UE, Scotland; c Department of Physics, Bhavan’s Sheth R. A. College of Science, Ahmedabad, Gujarat 380001, India; d Research Centre for Crystalline Materials, Faculty of Science and Technology, Sunway University, 47500 Bandar Sunway, Selangor Darul Ehsan, Malaysia

**Keywords:** crystal structure, salt, hydrogen bonding, mefloquine

## Abstract

The asymmetric unit of the title salt, C_17_H_17_F_6_N_2_O^+^·C_8_H_7_O_3_^−^·0.5H_2_O, comprises a pair of pseudo-enanti­omeric (*i.e*. related by a non-crystallographic centre of symmetry) piperidin-1-ium cations, two carboxyl­ate anions and a water mol­ecule of crystallization. The cations have similar conformations approximating to a letter, *L*: one of them shows disorder of its –CF_3_ group over two sets of sites in a 0.775 (3):0.225 (3) ratio. Distinctive conformations are found for the anions, one with the carboxyl­ate group lying to one side of the plane through the phenyl ring and the other where the oxygen atoms lie to either side of the plane. In the latter, an intra­molecular hy­droxy-O—H⋯O(carboxyl­ate) charge-assisted hydrogen bond is found. The packing features extensive O—H⋯O,N hydrogen bonding, often charge-assisted; C—H⋯π inter­actions are also formed. The hydrogen bonding results in the formation of five distinctive supra­molecular synthons and assembles mol­ecules in the *ac* plane. The quinolinyl rings lie to either side of the layer and inter-digitate with layers on either side, are approximately parallel to the *b* axis and are connected by π–π [inter-centroid separation = 3.6904 (18) Å] as well as C—F⋯π(quinolin­yl) inter­actions to consolidate the three-dimensional crystal. The dominance of the conventional hydrogen bonding in the mol­ecular packing is confirmed by an analysis of the Hirshfeld surface.

## Abstract 2

The l-shaped cations in the centrosymmetric title salt are related across a non-crystallographic centre of inversion. In the crystal, hydrogen-bonded layers are linked by π–π and C—H⋯F⋯π inter­actions.

## Chemical context   

When the racemic compound mefloquine is reacted with HCl, protonation occurs at the piperdinyl-N atom to yield the [(R*,S*)-(2-{[2,8-bis­(tri­fluoro­meth­yl)quinolin-4-yl](hy­droxymeth­yl)piperidin-1-ium chloride salt; see Scheme for the chemical diagram of the cation, also known as mefloqinium. This salt, racemic *erythro*-mefloquine hydro­chloride, has been used as an anti-malarial drug since 1971 (Maguire *et al.*, 2006 ▸). As an example of drug re-positioning, new biological activities have been sought for this drug and derivatives resulting in the disclosure of their potential as, for example anti-bacterial (Mao *et al.*, 2007 ▸), anti-mycobacterial (Gonçalves *et al.*, 2012 ▸) and anti-cancer (Rodrigues *et al.*, 2014 ▸) agents. This inter­est notwithstanding, it turns out that the crystal chemistry of the cation is rich and diverse. For example, the crystal structures of salts of the cation with three isomeric *n*-nitro­benzoates (*n* = 2, 3, and 4) have been described where the supra­molecular association led to chains in each case, but these were sustained by distinct hydrogen-bonded synthons (Wardell *et al.*, 2011 ▸).
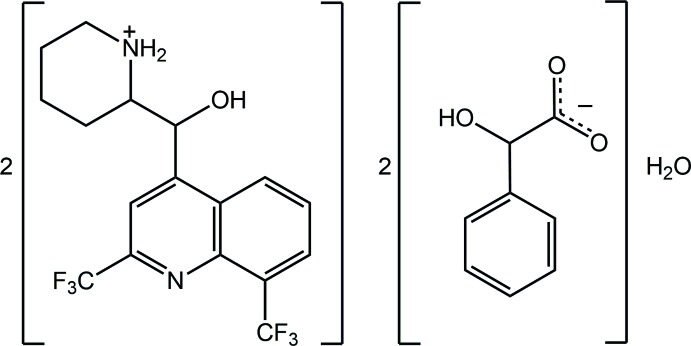


In addition, recently, two kryptoracemates have been revealed, namely in mefloqinium salts with *p*-fluoro­benzene­sulfonate (Jotani *et al.*, 2016 ▸) and (+)-3,3,3-tri­fluoro-2-meth­oxy-2-phenyl­propanate (Wardell *et al.*, 2016 ▸). It was in this context that the title hydrated salt, (I), was investigated: this was isolated after racemic mefloquine was reacted with a stoichiometric amount of racemic 2-hy­droxy-2-phenyl­acetic acid. Herein, the crystal and mol­ecular structures of the title salt, (I), are described as well as a Hirshfeld surface analysis.

## Structural commentary   

**Figure 1:**
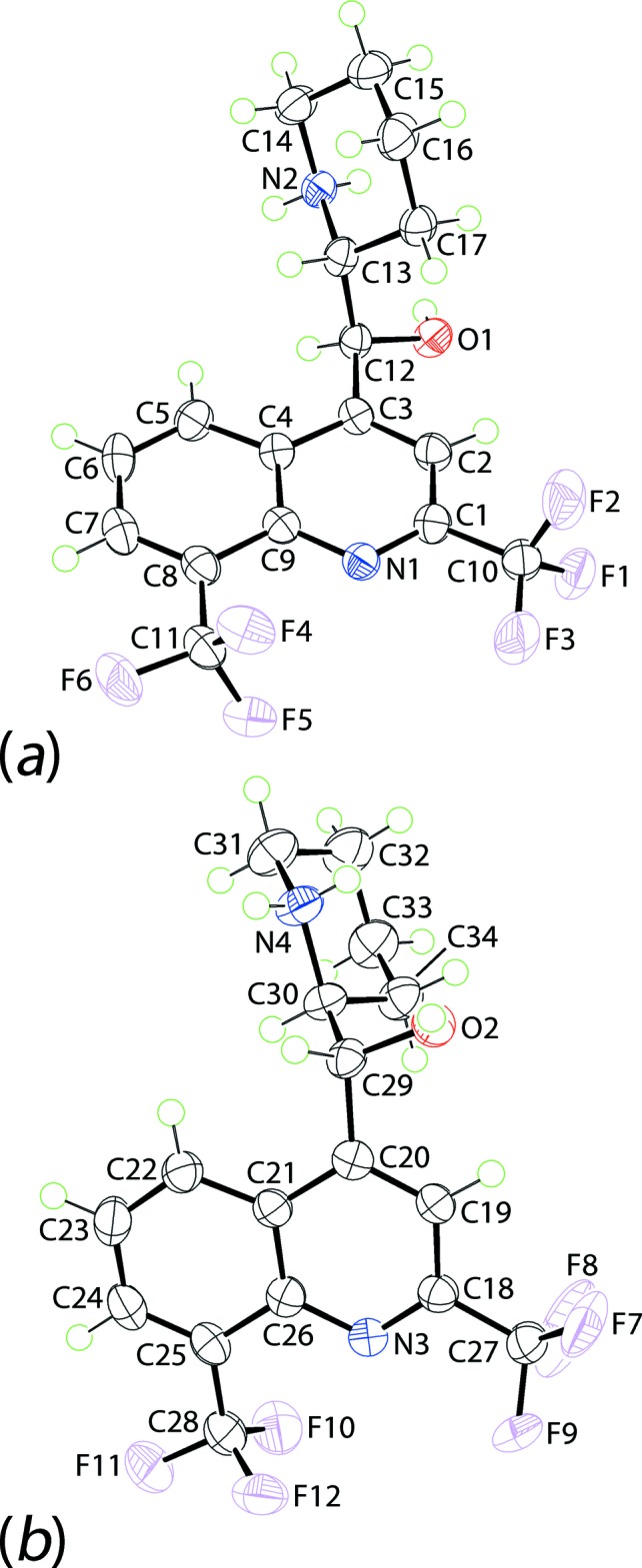
The mol­ecular structures of the (*a*) first and (*b*) second independent cations in (I) showing the atom-labelling scheme and displacement ellipsoids at the 70% probability level. For (*b*), only the major component of the disordered C27-CF_3_ group is shown.

**Figure 2:**
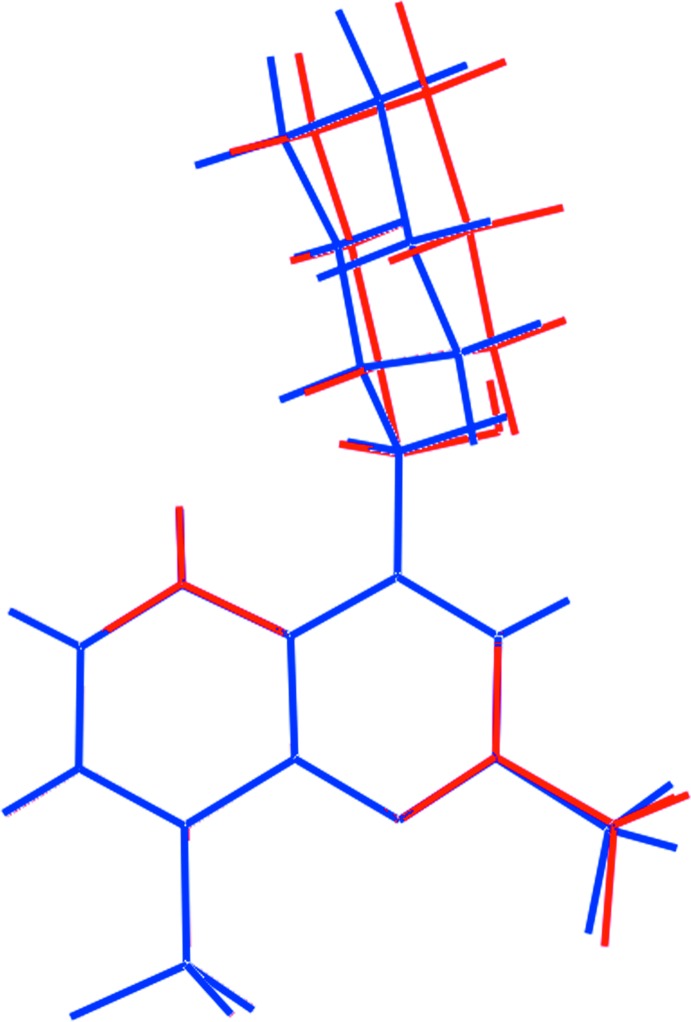
An overlap diagram highlighting the similarity of the conformations of the first (red) and inverted second (blue) independent cations. The cations have been overlapped so the the quinolinyl rings are coincident.

The asymmetric unit of (I) comprises two 2-{[2,8-bis­(trifluoro­meth­yl)quinolin-4-yl](hy­droxy)meth­yl}piperidin-1-ium cations, two 2-hy­droxy-2-phenyl­acetate anions and a water mol­ecule of crystallization. The cations, Fig. 1 ▸, are pseudo-enanti­omeric (*i.e*. related by a non-crystallographic inversion centre) with the N1-cation having an *S*-configuration at the C12 atom and an *R*-configuration at C13 and therefore being assigned as the [(−)-erythro-mefloquinium] cation. The N3-cation, with chirality at the C29 and C30 atoms being *R* and *S*, respectively, is assigned as [(+)-erythro-mefloquinium]. As anti­cipated, protonation during crystallization leads to a piperidin-1-ium cation, as confirmed by the pattern of hydrogen bonding, which is discussed below in *Supra­molecular features*. Each cation comprises an essentially planar quinolinyl residue attached to a piperidinium residue (with a chair conformation) *via* a methine link. The dihedral angle between the quinolinyl-NC_5_ ring plane and the best plane through the piperidinium ring is 71.91 (16)° indicating an almost perpendicular relationship so that the cation adopts an l-shape; the equivalent dihedral angle for the N3-cation is 80.58 (17)°. This assignment is also supported by the values of the C2—C3—C12—C13 and C19—C20—C29—C30 torsion angles of −100.4 (3) and 108.1 (3)°, respectively. The hydroxyl-O and piperidinium-N atoms lie to the same side of the piperidinium ring, being *gauche* across the methine-C—C(methine) bond with N2⋯O1 = 3.019 (4) Å and O1—C12—C13—N2 = 73.3 (3)° for the N1-cation; the equivalent values for the N3-cation are 2.931 (4) Å and −70.7 (3)°, respectively. The similarity in the two cations is emphasized in the overlay diagram shown in Fig. 2 ▸ where the inverted form of the N3-cation has been superimposed upon the N1-cation.

**Figure 3:**
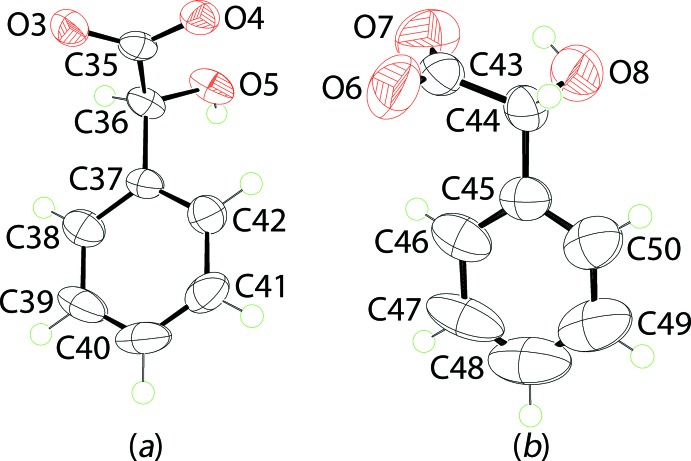
The mol­ecular structures of the (*a*) first and (*b*) second independent anions in (I) showing the atom-labelling scheme and displacement ellipsoids at the 70% probability level.

**Figure 4:**
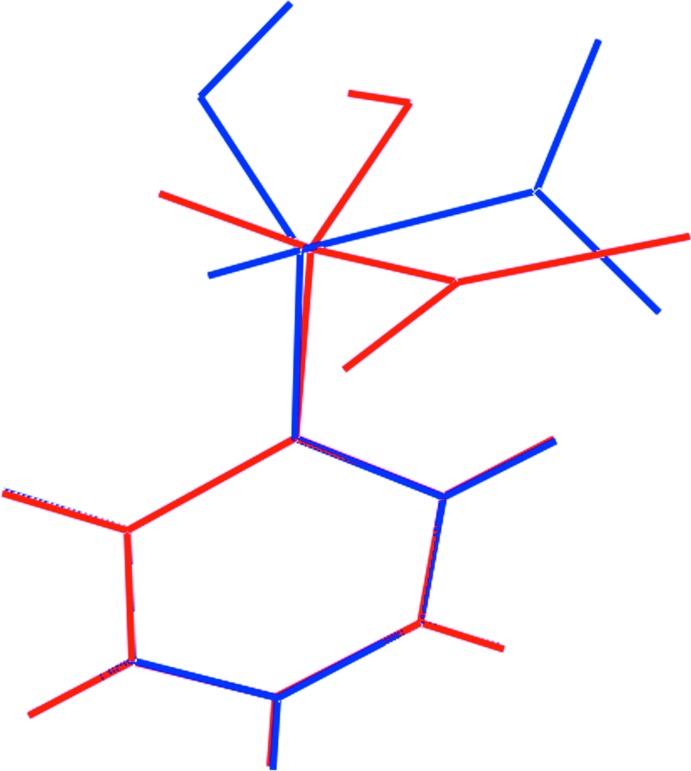
An overlap diagram highlighting the differences in the conformations of the first (red) and inverted second (blue) independent anions. The anions are overlapped so the phenyl rings are coincident.

The anions in (I), Fig. 3 ▸, were modelled with the N1-anion having an *S*-configuration at the C36 atom and an *R*-configuration at atom C44 of the second independent anion. The confirmation of deprotonation is found in the near equivalence of the C35—O3, O4 [1.260 (4) and 1.263 (4) Å] and of the C43—O6, O7 [1.223 (5) and 1.246 (5) Å] bond lengths. As evidenced from the overlay diagram shown in Fig. 4 ▸, which overlaps the inverted form of the O6-anion with the O3-anion with phenyl rings made coincident, major conformational differences between the anions exist. In the O3-anion, the dihedral angle between the phenyl ring and carboxyl­ate group is 71.2 (3)° which is a little more acute than the comparable angle of 78.4 (4)° for the O6-anion. However, the significant difference arises in the relative dispositions of the carboxyl­ate group to the phenyl ring, lying completely to one side of the ring for the O3-anion but with one carboxyl­ate-O atom above and the other below the plane through the phenyl ring for the O6-anion. This difference is qu­anti­fied in the disparity in the C35—C36—C37—C38 and C43—C44—C45—C46 torsion angles of 108.0 (3) and 20.0 (6)°, respectively. Another difference is noted in the formation of an intra­molecular hy­droxy-O—H⋯O(carboxyl­ate) hydrogen bond in only one of the anions. In both cases the hy­droxyl O atoms is to a first approximation *syn* to a carboxyl­ate-O atom as seen in the O3—C35—C36—O5 and O7—C43—C44—O8 torsion angles of 151.9 (3) and 17.3 (6)°, respectively. However, it is only in the O6-anion that the aforementioned hydrogen bond is formed to close a five-membered {⋯HOC_2_O} loop, Table 1 ▸.

## Supra­molecular features   

In addition to considerable conventional hydrogen bonding, often charge-assisted, there are other inter­molecular inter­actions at play in the mol­ecular packing (Spek, 2009 ▸). The geometric parameters characterizing most of these inter­molecular inter­actions are given in Table 1 ▸. The pattern of hydrogen bonding clearly differentiates both the cations and in the same way, the anions. Thus, the hy­droxy group of the N1-cation forms a charge-assisted hy­droxy-O—H⋯O(carboxyl­ate) inter­action with an anion, while the hydroxyl group of the N3-cation forms a hy­droxy-O—H⋯O(hy­droxy) link between the cations. The piperidinium-N—H_2_ H atoms of the N1-cation forms charge-assisted hydrogen bonds to the water mol­ecule of crystallization and to the O3-carboxyl­ate atom, whereas those of the N3-cation inter­act with the hy­droxy-O8 and carboxyl­ate-O4 atoms.

A different hydrogen-bonding pattern is also noted for the anions, already differentiated by the formation of an intra­molecular hy­droxy-O—H⋯O(carboxyl­ate) inter­action in the O6-anion. The hy­droxy group of the O3-anion forms a hy­droxy-O—H⋯O(carboxyl­ate) link between the anions. Both carboxyl­ate-O3, O4 atoms accept hydrogen bonds from piperidinium-N—H H atoms whereas the carboxyl­ate-O5, O6 atoms form inter­actions with piperidinium-N—H and anion–hydroxyl-H H atoms, respectively. The carboxyl­ate-O3 and O7 atoms each form two hydrogen bonds with the additional inter­actions involving water-H atoms. Finally, as just mentioned, the water mol­ecule forms two donor inter­actions with carboxyl­ate-O atoms, accepts a hydrogen bond from a piperidinium-N—H H atom and also accepts a contact from a quinolinyl-C—H atom.

**Figure 5:**
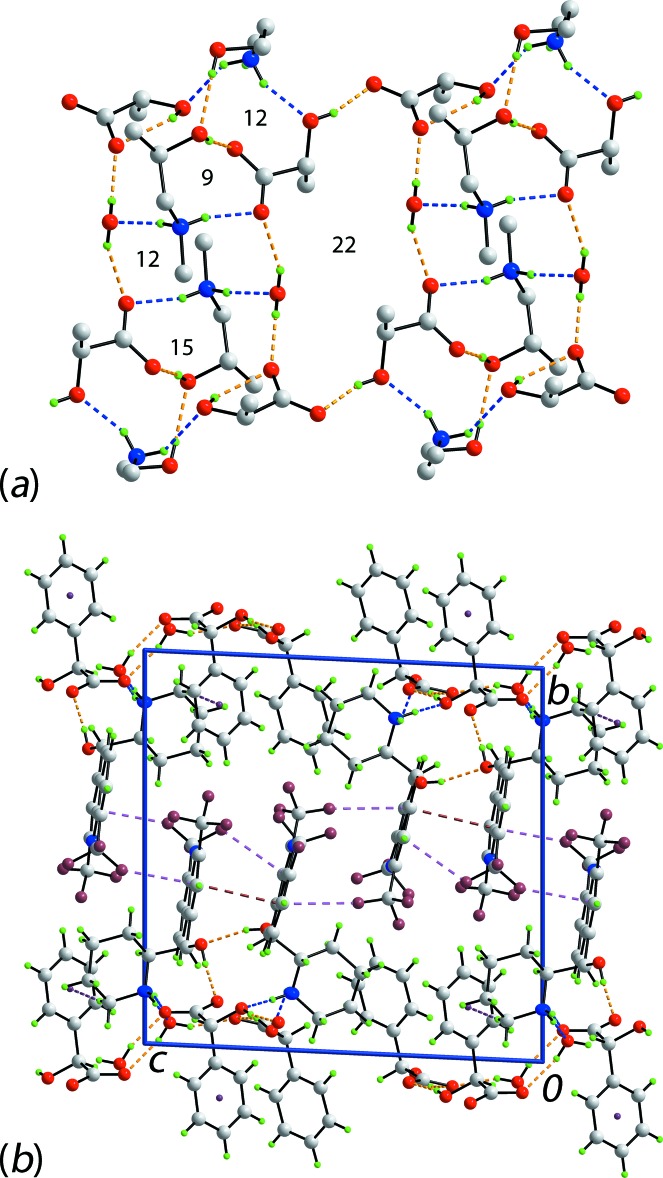
The mol­ecular packing in (I): (*a*) a portion of the hydrogen bonding highlighting the formation of supra­molecular synthons and (*b*) a view in projection down the *a* axis of the unit-cell contents. The O—H⋯O and N—H⋯O hydrogen bonds are shown as orange and blue dashed lines, respectively, and the C—H⋯π, π—π and C—F⋯π inter­actions are shown as purple, brown and pink dashed lines, respectively. Colour code: F, cyan; O, red; N, blue; C, grey; and H, green.

The just described hydrogen bonding gives rise to a number of cyclic synthons. Referring to Fig. 5 ▸ *a*, the largest synthon is sustained exclusively by O—H⋯O hydrogen bonding, being a centrosymmetric 22-membered {⋯OCO⋯HOH⋯OC_2_OH}_2_ ring. Four other rings are formed mediated by hydrogen bonding but the only remaining centrosymmetric synthon features two bridging piperidinium-N—H H atoms, which link water- and carboxyl­ate-O atoms to generate a 12-membered {⋯HNH⋯OH⋯O}_2_ synthon. The three remaining synthons do not have symmetry. The smallest, nine-membered {⋯HNC_2_OH⋯OCO} abuts the 12-membered synthon just described and shares a common N—H bond. The nine-membered synthon is connected on the other side by an 12-membered ring featuring the second piperidinium-N—H_2_ group, *i.e*. {⋯HNC_2_OH⋯OH⋯OC_2_O}. Portions of both of the nine- and 12-membered synthons participate in the formation of a larger 15-membered synthon which involves both piperidinium-N—H_2_ groups, *i.e*. {⋯HNH⋯OC_2_O⋯HNH⋯OH⋯O⋯HO}; one of the O—H⋯O links is the intra­molecular hy­droxy-O—H⋯O(carboxyl­ate) hydrogen bond. A tight methyl­ene-C—H⋯π(anion-phen­yl) inter­action is also noted, Table 1 ▸. The hydrogen bonding extends laterally in the *ac* plane with the quinolinyl residues lying to either side in the *b-*axis direction and in orientations enabling inter-digitation. Inter­actions between rings are of the type π–π, occurring between quinolinyl-bound (C21–C26) and (N1,C1–C4,C9)^i^ rings with an inter-centroid separation of 3.6904 (18) Å and angle of inclination of 8.70 (15)°; symmetry code (i): 1 − *x*, 1 − *y*, 1 − *z*. A variety of C—F⋯π(quinolin­yl) inter­actions provide additional links in the inter-layer region. A view of the unit-cell contents is shown in Fig. 5 ▸ *b*.

## Hirshfeld surface analysis   

*Crystal Explorer* (Wolff *et al.*, 2012 ▸) was used to generate Hirshfeld surfaces mapped over *d*_norm_, shape-index, curvedness and electrostatic potential. The electrostatic potentials were calculated using *TONTO* (Spackman *et al.*, 2008 ▸; Jayatilaka *et al.*, 2005 ▸) integrated into *Crystal Explorer*; the crystal geometry was used as the input. The electrostatic potentials were mapped onto Hirshfeld surfaces using the STO-3G basis set at the Hartree–Fock level of theory. The contact distances *d*_i_ and *d*_e_ from the Hirshfeld surface to the nearest atom inside and outside, respectively, enables the analysis of the inter­molecular inter­actions through the mapping of *d*_norm_.

**Figure 6:**
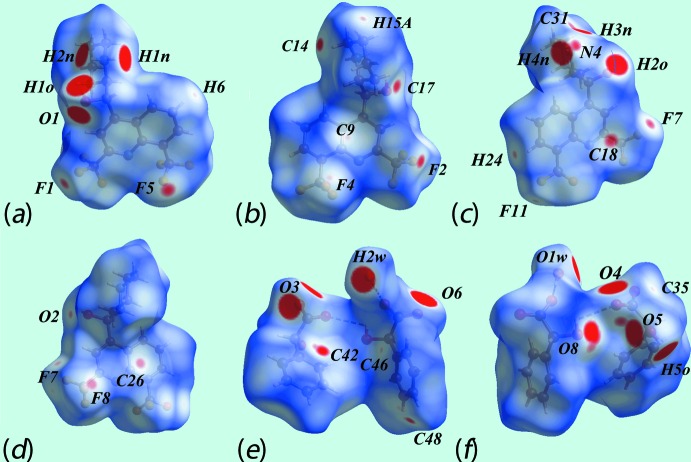
Views of Hirshfeld surfaces mapped over *d*_norm_ for (*a*) and (*b*) the N1-cation, (*c*) and (*d*) the N3-cation and (*e*) and (*f*) the anions and water mol­ecule.

**Figure 7:**
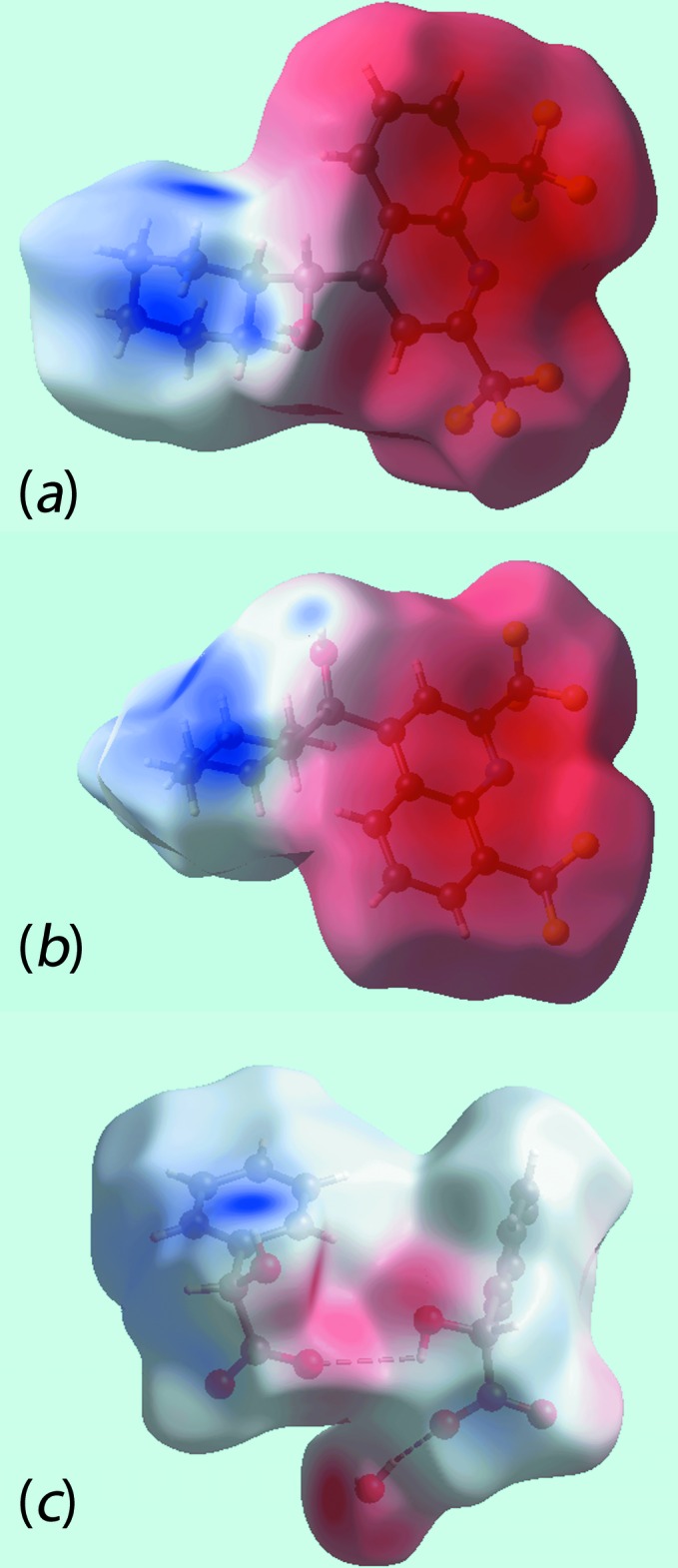
View of Hirshfeld surfaces mapped over electrostatic potential for (*a*) the N1-cation (*b*) the N3-cation and (*c*) the anions and water mol­ecule.

The Hirshfeld surfaces mapped over *d*_norm_ for the N1-cation, N3-cation and the entity comprising the two anions together with the water mol­ecule of crystallization are illustrated in Fig. 6 ▸, and Hirshfeld surfaces mapped over electrostatic potential for the same species, in the ranges −0.25 to +0.17, −0.26 to +0.17 and −0.14 to +0.20 au, respectively, are illustrated in Fig. 7 ▸. The mapping of Hirshfeld surfaces over *d*_norm_ in the range −0.5 to +1.3 au reveals potential hydrogen-bond donors and acceptors as bright-red spots. The further mapping of Hirshfeld surfaces over *d*_norm_ in the range −0.1 to +1.1 au results in faint-red spots on the surfaces which can satisfactorily describe the influence of other inter­molecular inter­actions in the crystal such as C—H⋯O, C—H⋯F, C—H⋯π, C—F⋯π and π–π stacking. The bright-red spots appearing near the donor hydroxyl-H2*O*, Fig. 6 ▸ *c*, and acceptor hydroxyl-O1*O* atom, Fig. 6 ▸ *a*, show the O—H⋯O link between the two independent cations. The charge-assisted O—H⋯O inter­action between the hydroxyl-H1*O* and carboxyl­ate-O4 atoms can be viewed as bright-red spots in Fig. 6 ▸ *a* and 6*f*, respectively. The bright-red spots at the piperidinium-H1*N* and H2*N* atoms, Fig. 6 ▸ *a*, and oxygen atoms O3, Fig. 6 ▸ *e*, and O1*W*, Fig. 6 ▸ *f*, indicate the formation of N—H⋯O hydrogen bonds associated with the N1-cation. The other group of N—H⋯O bonds resulted from piperidinium-H3*N* and H4*N* of the N3-cation and are apparent as the bright-red spots on the surface donors, Fig. 6 ▸ *c*, and acceptors, Fig. 6 ▸ *f* (*i.e*. carboxyl­ate O4 and O8), respectively; the faint-red spots near the piperidinium-N4, Fig. 6 ▸ *c*, and carboxyl­ate-O4 atoms, Fig. 6 ▸ *e*, are due to the presence of comparatively weak N—H⋯O hydrogen bonds. The existence of water-O—H⋯O hydrogen bonds can be viewed as bright-red spots near the H2*W* and carboxyl­ate-O3 atoms while the other is indicated with dashed lines in Fig. 6 ▸ *e*. Finally, the bright-red spots at hydroxyl-H5*O*, Fig. 6 ▸ *f*, and carboxyl­ate-O6, Fig. 6 ▸ *e*, provides a link between the anions through O—H⋯O inter­actions.

**Figure 8:**
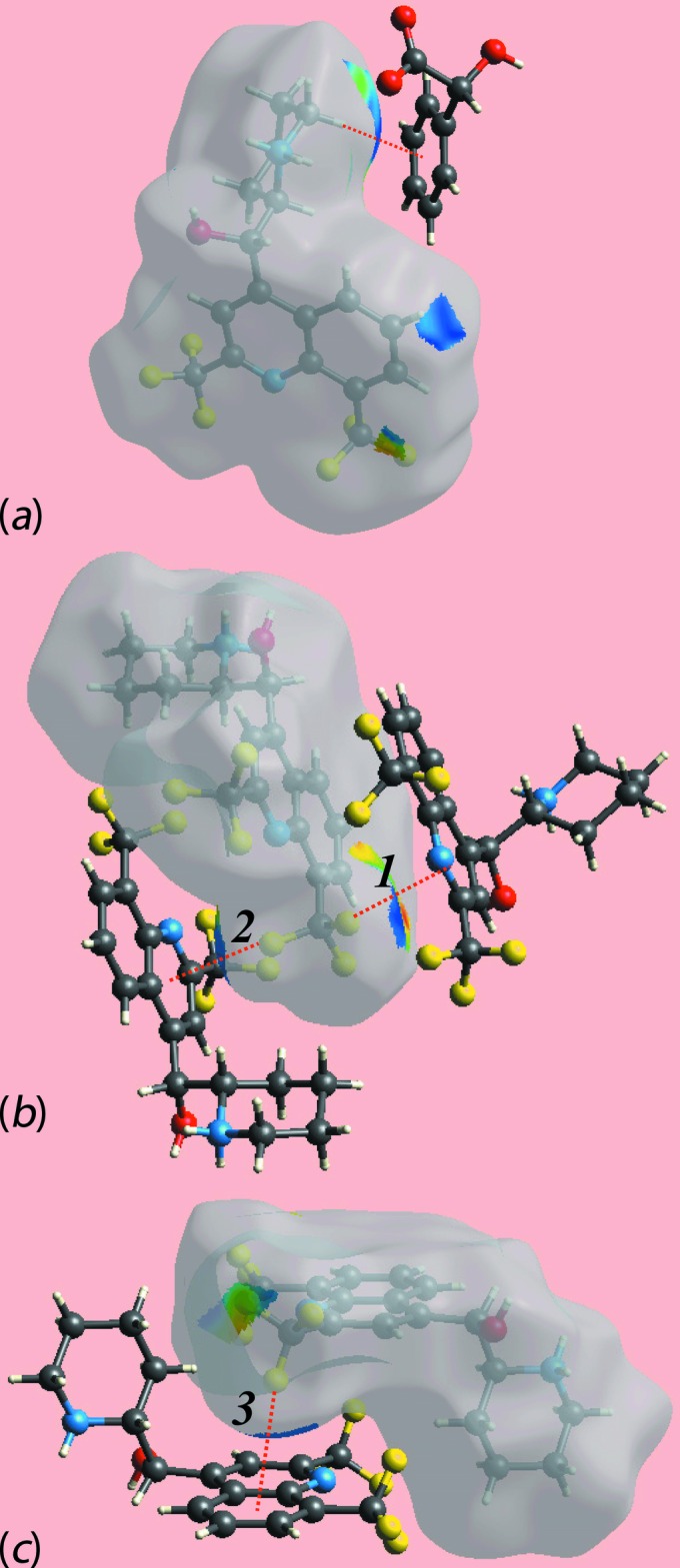
Views of Hirshfeld surfaces mapped over the shape-index showing (*a*) C—H⋯π, (*b*) and (*c*) C—F⋯π inter­actions. The inter­actions are indicated with red-dotted lines.

**Figure 9:**
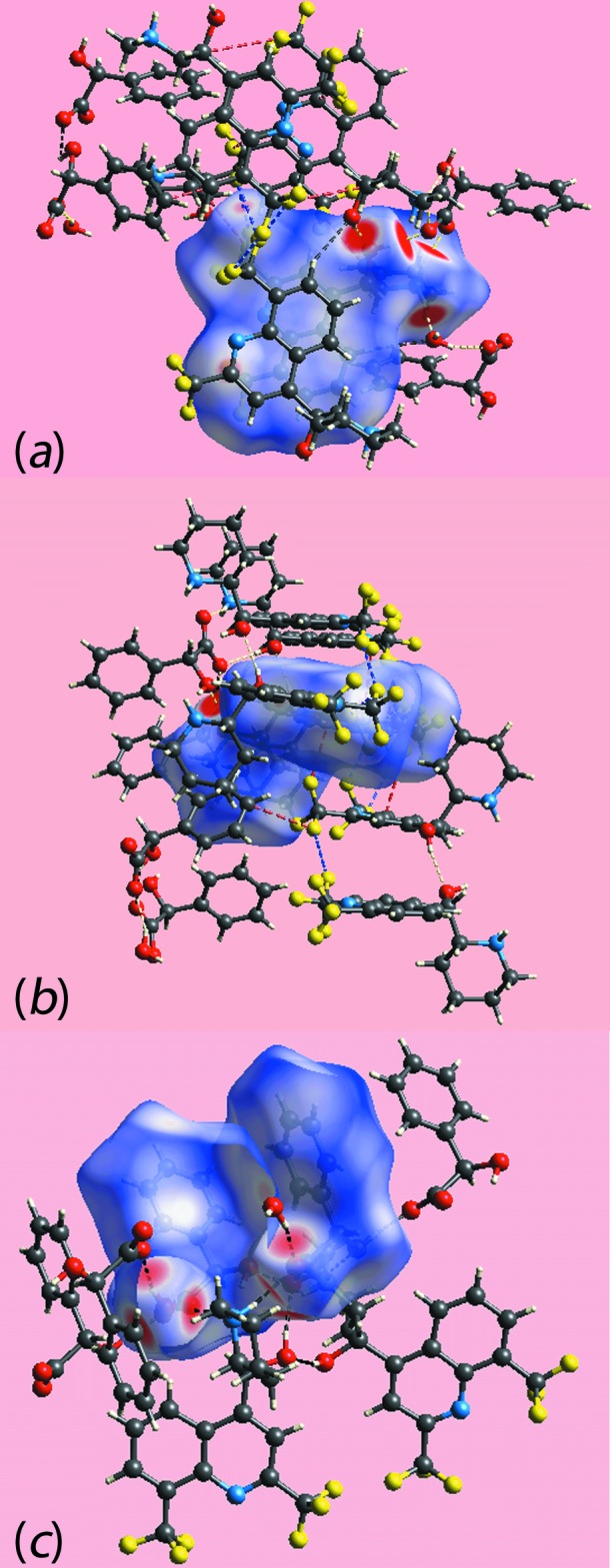
The immediate environments about the (*a*) N1-cation, (*b*) N3-cation and (*c*) anions and water mol­ecule. The reference mol­ecule within the Hirshfeld surfaces are mapped over *d*_norm_ and highlight their participation in inter­molecular inter­actions.

The faint-red spots near the fluorine atoms of the CF_3_ groups of the cations indicate their participation in various inter­molecular inter­actions. The faint-red spots near the F1, F7 and F11 atoms shown in Figs. 6 ▸ *a*, 6*c* and 6*d*, indicate short inter­atomic F⋯F contacts, Table 2 ▸. The spots near the F2 and piperidinium-C17 atoms arise form inter­molecular C—H⋯F inter­actions, Fig. 6 ▸ *b* and Table 2 ▸. The presence of C—F⋯π inter­actions are evident from the diminutive-red spots near the F4 and F5 atoms of the N1-cation, and F8 of the N3-cation, Figs. 6 ▸ *a*, 6*b* and 6*d*, and from the short inter­atomic C⋯F contacts listed in Table 2 ▸. The Hirshfeld surfaces mapped with shape-index properties are illustrated in Fig. 8 ▸ and reflect these C—F⋯π inter­actions. In addition to above, the short inter­atomic C48⋯F7 contact is also viewed as very faint-red spots near these atoms on the surface, Figs. 6 ▸ *c*, 6*d* and 6*e*. The faint-red spots present near the methyl­ene-C14—H, Fig. 6 ▸ *b*, and anion-phenyl-C42 atoms, Fig. 6 ▸ *e*, and short inter­atomic C⋯H/H⋯C contacts between methyl­ene-H14*A* and anion atoms C37, C41 and C42, as summarized in Table 2 ▸, clearly indicate their contribution to the C—H⋯π inter­action described above. The presence of a C—H⋯O inter­action between piperidinium-C31—H of the N3-cation and hydroxyl-O8 of one of the anions is observed as diminutive-red spots near these atoms in Figs. 6 ▸ *c* and 6*f*, and qu­anti­fied in Table 2 ▸. In addition to the above inter­molecular inter­actions related to C⋯H/H⋯C contacts, the short inter­atomic contacts between the anion-C46 and C35 atoms, Figs. 6 ▸ *e* and 6*f*, and N1-cation hydrogens H6 and H15*A*, Figs. 6 ▸ *a* and 6*b*, are also viewed as faint-red spots near these atoms. The immediate environments about the N1- and N3-cations and the anions and water mol­ecule within the *d*_norm_-mapped Hirshfeld surface mediated by the above inter­actions are illustrated in Fig. 9 ▸.

**Figure 10:**
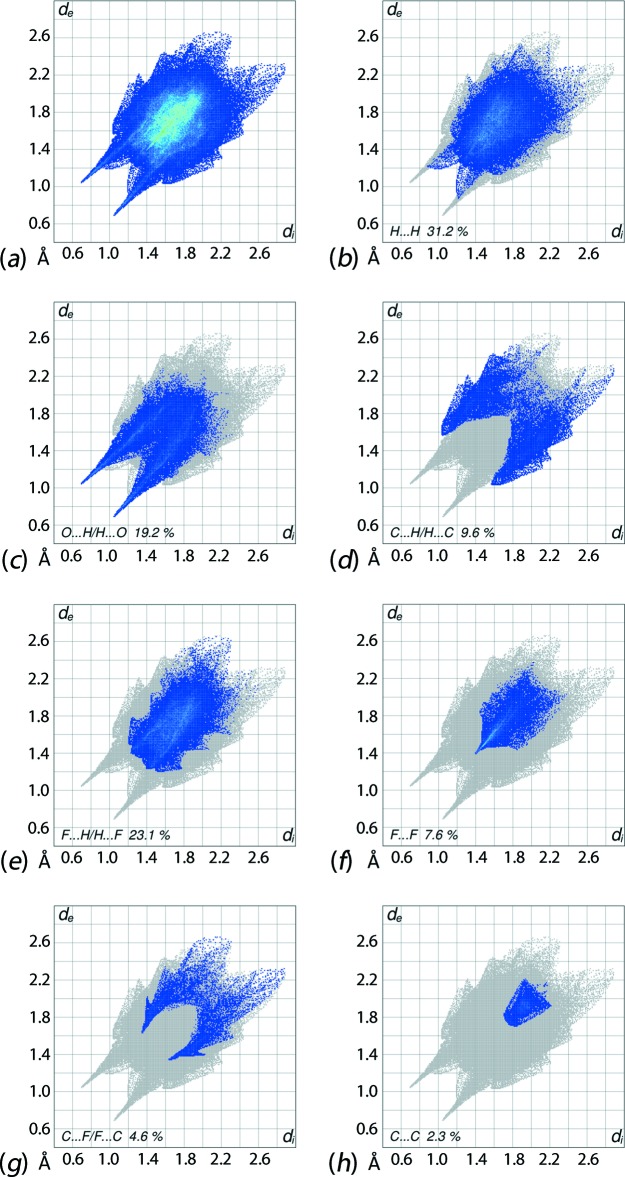
Two-dimensional fingerprint plots calculated for (I): (*a*) overall plot, and plots delineated into (*b*) H⋯H, (*c*) O⋯H/H⋯O, (*d*) C⋯H/H⋯C, (*e*) F⋯H/H⋯F, (*f*) F⋯F, (*g*) C⋯F/F⋯C and (*h*) C⋯C contacts.

The combination of *d*_i_ and *d*_e_ in the form of two-dimensional fingerprint plots (McKinnon *et al.*, 2007 ▸) provides a summary of the inter­molecular contacts occurring in the crystal. The overall two-dimensional fingerprint plot for (I) and those delineated into H⋯H, O⋯H/H⋯O, C⋯H/H⋯C, F⋯H/H⋯F, F⋯F, C⋯F/F⋯C and C⋯C contacts (McKinnon *et al.*, 2007 ▸) are illustrated in Fig. 10 ▸ *a*–*h*, respectively; their relative contributions are summarized in Table 3 ▸. The fingerprint plot delineated into H⋯H contacts, Fig. 10 ▸ *b*, shows that although these make the greatest contribution to the overall Hirshfeld surface, *i.e*. 31.2%, its comparatively low value is due to the involvement of many of the available hydrogen atoms of the various functional groups in specific inter­molecular O—H⋯O and N—H⋯O hydrogen bonds. A nearly symmetric (mirror) distribution of points reflected as a saw-tooth with the tips at *d*_e_ + *d*_i_ ∼2.1 Å correspond to a short inter­atomic piperidinium-H1*O*⋯H2*O* contact between hydroxyl-hydrogens of the two independent cations, Table 2 ▸; the other short inter­atomic H⋯H contacts, Table 2 ▸, are associated with the points distributed in (*d*_e_, *d*_i_) region less than the van der Waals separations, *i.e*. 2 × 1.2 Å. The 19.2% contributions from O⋯H/H⋯O contacts to the overall surface results from inter­molecular O—H⋯O, N—H⋯O and C—H⋯O inter­actions as well as short inter­atomic O⋯H/H⋯H contacts in the crystal, Table 2 ▸. In the fingerprint plot delineated into O⋯H/H⋯O contacts, Fig. 10 ▸ *c*, a pair of long spikes having tips at *d*_e_ + *d*_i_ ∼1.7 Å and the appearance of green points aligned as a pair of streaks are due to the presence of dominant O—H⋯O and N—H⋯O hydrogen bonds.

**Figure 11:**
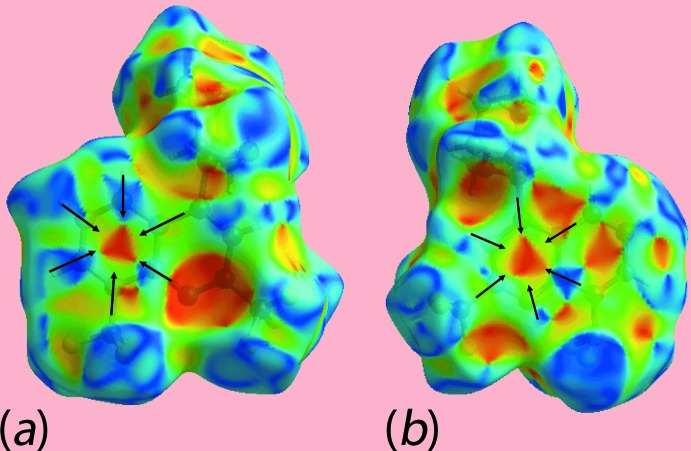
Views of Hirshfeld surfaces mapped over the shape-index for the (*a*) (N1,C1–C3,C9) and (*b*) (C21–C26) rings, highlighting π–π stacking.

**Figure 12:**
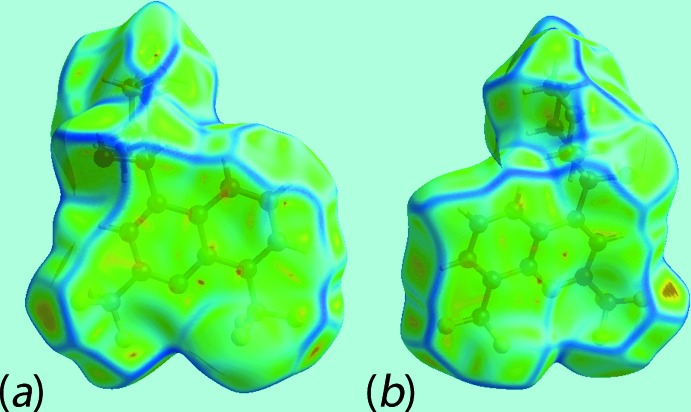
Views of Hirshfeld surfaces mapped over the curvedness for the (*a*) (N1,C1–C3,C9) and (*b*) (C21–C26) rings, highlighting π–π stacking.

The fingerprint plot corresponding to C⋯H/H⋯C contacts, Fig. 10 ▸ *d*, show a fin-like distribution of points with the edges at *d*_e_ + *d*_i_ ∼2.6 Å resulting from the presence of C—H⋯π inter­actions and short inter­atomic C⋯H/H⋯C contacts, as summarized in Table 2 ▸. The presence of a pair of two small peaks at *d*_e_ + *d*_i_ ∼2.7 Å and 2.8 Å in a tube-shaped distribution of points in the fingerprint plot delineated into F⋯H/H⋯F contacts, Fig. 10 ▸ *e*, arise from short inter­molecular F⋯H/H⋯F contacts, Table 2 ▸. The presence of two tri­fluoro­methyl groups in each cation increases the percentage contribution from these contacts to the Hirshfeld surface to 23.1%, thereby contributing to the reduced relative contribution from H⋯H contacts. In the fingerprint delineated into F⋯F contacts, Fig. 10 ▸ *f*, the distribution of points in a pencil-tip shape with the tip at *d*_e_ + *d*_i_ ∼2.8 Å represent the short inter­atomic F⋯F contacts listed in Table 2 ▸. The inter­molecular C—F⋯π and C⋯F inter­actions in the crystal are characterized by a fin-shape, at *d*_e_ + *d*_i_ ∼3.0 Å, in the fingerprint plot delineated into C⋯F/F⋯C contacts, Fig. 10 ▸ *g*, and make a 4.6% contribution to the surface. A small 2.3% contribution from C⋯C contacts to the Hirshfeld surface with the parabolic distribution of points, Fig. 10 ▸ *h*, around the (*d*_e_, *d*_i_) distances slightly shorter than their van der Waals radii, *i.e*. 2 × 1.7 Å, indicate π–π stacking inter­actions between quinolinyl rings. The presence of π–π stacking inter­actions between the symmetry-related rings is also indicated through the appearance of red and blue triangle pairs on the Hirshfeld surface mapped with shape-index property identified with arrows in the images of Fig. 11 ▸, and in the flat regions on the Hirshfeld surfaces mapped over curvedness in Fig. 12 ▸.

## Database survey   

Recent contributions to the structural chemistry of mefloqinium salts (Jotani *et al.*, 2016 ▸; Wardell *et al.*, 2016 ▸) have included tabulated summaries of related literature structures and key geometric parameters. The cations in (I) conform to expectation. Two recently determined structures are particularly noteworthy as they exhibit kryptoracemic behaviour, *i.e*. contain enanti­omeric species that are not related by crystallographic symmetry, meaning they crystallize in one of the 65 Sohncke space groups, which lack inversion centres, rotatory inversion axes, glide planes and mirror planes. This phenomenon is rare for organic species, occurring in just 0.1% of their structures (Fábián & Brock, 2010 ▸). The two kryptoracemates arise for different reasons. In the first example, the ortho­rhom­bic (*P*2_1_2_1_2_1_) crystals isolated from the 1:1 reaction of mefloquinium chloride and *p*-fluoro­benzene­sulfonyl chloride in the presence of NaOH (Jotani *et al.*, 2016 ▸), contained [(+)-*erythro*-mefloquinium] and [(−)-*erythro*-mefloquinium] cations as well as a chloride and *p*-fluoro­benzene­sulfonate anions to provide the charge balance. The second example was isolated from the attempted chiral resolution of mefloquine with the carb­oxy­lic acid, 3,3,3-tri­fluoro-2-meth­oxy-2-phenyl­propanoic acid, *i.e*. (+)-PhC(CF_3_)(OMe)CO_2_H. Crystallography showed the triclinic (*P*1) crystals to comprise the [(+)-*erythro*-mefloquinium] and [(−)-*erythro*-mefloquinium] cations with two independent (+)-3,3,3-tri­fluoro-2-meth­oxy-2-phenyl­propanate anions. Hence, different anions appear to have promoted kryptoracemic behaviour in the chloride/*p*-fluoro­benzene­sulfonate salt (Jotani *et al.*, 2016 ▸) and distinctive crystal packing is responsible for this behaviour in the (+)-3,3,3-tri­fluoro-2-meth­oxy-2-phenyl­propanate salt (Wardell *et al.*, 2016 ▸). The latter reason seems to apply in the case of (I) where a non-crystallographic symmetry relationship exists between the cations. However, (I) being centrosymmetric indicates that kryptoracemic-type behaviour for the mefloquinium cation is not limited to non-centrosymmetric structures.

## Synthesis and crystallization   

Solutions of mefloquine (1 mmol) in MeOH (15 ml) and (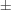)PhCHOHCO_2_H (1 mmol) in MeOH (10 ml) were mixed at room temperature. The reaction mixture was set aside at room temperature for three days and the resulting colourless slabs collected; M.pt: 434–346 K. IR (KBr disc): 3400–2100 (*v br*), 1586, 1313, 1190, 1130, 739 cm^−1. 13^C NMR (100 MHz, *d*_6_-DMSO): δ 21.74, 22.01, 22.38, 44.54, 59.25, 68.39, 73.45, 115.43, 121.22 [*J*(C—F) = 273.6 Hz], 123.69 [*J*(C—F) = 272.2 Hz], 126.34, 126.38, 126.52, 127.19 [*J*(C—F) = 291.3 Hz], 127.53, 128.11. 129.05, 129.76 [*J*(C—F) = 4.7 Hz], 142.74, 143.03, 146.64 [*J*(C—F) = 34. 0 Hz], 151.82, 175.75 p.p.m.

## Refinement   

Crystal data, data collection and structure refinement details are summarized in Table 4 ▸. The H atoms were geometrically placed (C—H = 0.95–1.00 Å) and refined as riding with *U*_iso_(H) = 1.2*U*_eq_(C). The O- and N-bound H atoms were located from difference maps but, refined with O—H = 0.84±0.01 Å and N—H = 0.88±0.01 Å, and with *U*_iso_(H) = 1.2*U*_eq_(N) and 1.5*U*_eq_(O). One reflection, *i.e*. (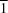 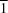
1), was omitted from the final refinement owing to poor agreement. The C27-CF_3_ group was modelled as being disordered over two orientations with a site occupancy ratio 0.775 (3):0.225 (3). The anisotropic displacement parameters for pairs of F atoms were constrained to be equal and restrained to be nearly isotropic. Even so, one atom in particular showed elongated displacement ellipsoids, *i.e*. the F8 atom, but this was not modelled further. Multiple atomic positions were not discerned for the O6-anion, Fig. 3 ▸ *b*. Finally, the maximum and minimum residual electron density peaks of 1.75 and 0.66 eÅ^−3^, respectively, were located 0.84 Å and 0.35 Å from the H44 and O7 atoms, respectively. Given the strong and directional hydrogen bonding in this region of the mol­ecule, it is likely that the large residual is an artefact of the data.

## Supplementary Material

Crystal structure: contains datablock(s) I, global. DOI: 10.1107/S2056989016016492/hb7620sup1.cif

Structure factors: contains datablock(s) I. DOI: 10.1107/S2056989016016492/hb7620Isup2.hkl

Supporting information file. DOI: 10.1107/S2056989016016492/hb7620Isup3.cml

CCDC reference: 1510084

Additional supporting information: crystallographic information; 3D view; checkCIF report

## Figures and Tables

**Table 1:** Hydrogen-bond geometry (Å, °) *Cg*1–*Cg*4 are the ring centroids of the (C37–C42), (N1,C1–C4,C9), (N3,C18–C21,C26) and (C21–C26) rings, respectively.

*D*—H⋯*A*	*D*—H	H⋯*A*	*D*⋯*A*	*D*—H⋯*A*
O8—H8*O*⋯O7	0.86 (5)	2.00 (6)	2.638 (5)	131 (5)
O1—H1*O*⋯O4^i^	0.84 (2)	1.76 (3)	2.597 (3)	173 (4)
O2—H2*O*⋯O1^ii^	0.84 (2)	1.95 (2)	2.779 (3)	169 (5)
N2—H1*N*⋯O1*W* ^i^	0.89 (3)	1.85 (3)	2.725 (4)	167 (3)
N2—H2*N*⋯O3^i^	0.88 (2)	1.93 (2)	2.788 (4)	165 (3)
N4—H3*N*⋯O8^iii^	0.88 (2)	2.18 (3)	2.798 (5)	127 (3)
N4—H4*N*⋯O4^iii^	0.88 (3)	2.43 (3)	3.059 (4)	129 (3)
N4—H4*N*⋯O5^iii^	0.88 (3)	1.90 (3)	2.727 (4)	156 (3)
O5—H5*O*⋯O6^iv^	0.85 (3)	1.74 (3)	2.572 (4)	165 (5)
O1*W*—H1*W*⋯O7	0.84 (2)	1.84 (2)	2.635 (4)	156 (5)
O1*W*—H2*W*⋯O3^v^	0.84 (3)	1.98 (4)	2.768 (3)	156 (5)
C5—H5⋯O1*W* ^i^	0.95	2.59	3.539 (4)	175
C14—H14*A*⋯*Cg*1^vi^	0.99	2.66	3.642 (4)	171
C11—F4⋯*Cg*2^vii^	1.35 (1)	2.93 (1)	4.118 (3)	146 (1)
C11—F5⋯*Cg*3^viii^	1.34 (1)	3.15 (1)	3.931 (3)	117 (1)
C27—F8⋯*Cg*4^ii^	1.26 (1)	3.23 (1)	4.474 (3)	170 (1)

Symmetry codes: (i) 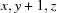; (ii) 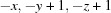; (iii) 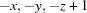; (iv) 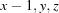; (v) 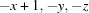; (vi) 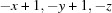; (vii) 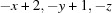; (viii) 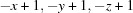.

**Table 2:** Additional inter­atomic contacts (Å) in the crystal of (I)

Parameter	Distance	Symmetry operation
F1⋯F7	2.787 (5)	−*x*, 1 − *y*, 1 − *z*
F7⋯F11	2.871 (4)	−1 + *x*, *y*, *z*
F2⋯C17	3.029 (4)	1 − *x*, 1 − *y*, −*z*
F2⋯H17*B*	2.65	1 − *x*, 1 − *y*, −*z*
F10⋯H34*A*	2.63	−*x*, 1 − *y*, 1 − *z*
F4⋯C9	3.153 (3)	2 − *x*, 1 − *y*, −*z*
F5⋯C18	2.971 (4)	1 − *x*, 1 − *y*, 1 − *z*
F8⋯C26	3.054 (5)	−*x*, 1 − *y*, 1 − *z*
F7⋯C48	3.148 (2)	−*x*, 1 − *y*, 1 − *z*
H14*A*⋯C37	2.74	1 − *x*, 1 − *y*, 1 − *z*
H14*A*⋯C41	2.88	1 − *x*, 1 − *y*, 1 − *z*
H14*A*⋯C42	2.61	1 − *x*, 1 − *y*, 1 − *z*
C31⋯O8	3.103 (5)	−*x*, −*y*, 1 − *z*
H31*A*⋯O8	2.62	−*x*, −*y*, 1 − *z*
C35⋯H6	2.74	−1 + *x*, −1 + *y*, *z*
C46⋯H15*A*	2.72	1 − *x*, 1 − *y*, −*z*
H1*O*⋯H2*O*	2.10 (5)	*x*, 1 − *y*, 1 − *z*
H1*N*⋯H1*W*	2.25	*x*, 1 + *y*, *z*
H1*N*⋯ H2*W*	2.24	*x*, 1 + *y*, *z*
H3*N*⋯H8*O*	2.32 (6)	−*x*, −*y*, 1 − *z*
H4*N*⋯H5*O*	2.38 (5)	−*x*, −*y*, 1 − *z*
O5⋯H22	2.65	−*x*, −*y*, 1 − *z*
O5⋯H29	2.67	−*x*, −*y*, 1 − *z*
O2⋯H24	2.50	1 + *x*, *y*, *z*

**Table 3:** Percentage contributions of different inter­atomic contacts to the Hirshfeld surface in (I)

Contact	%
H⋯H	31.2
O⋯H/H⋯O	19.2
F⋯H/H⋯F	23.1
C⋯H/H⋯C	9.6
C⋯F/F⋯C	4.6
F⋯F	7.6
C⋯C	2.3
F⋯N/N⋯F	1.4
C⋯N/N⋯C	0.7
N⋯H/H⋯N	0.3

**Table 4:** Experimental details

Crystal data	
Chemical formula	2C_17_H_17_F_6_N_2_O^+^·2C_8_H_7_O_3_^−^·H_2_O
*M*_r_	1078.95
Crystal system, space group	Triclinic, *P* 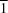
Temperature (K)	120
*a*, *b*, *c* (Å)	9.5317 (2), 15.8217 (5), 16.2980 (5)
α, β, γ (°)	85.926 (2), 77.418 (2), 83.003 (2)
*V* (Å^3^)	2378.46 (12)
*Z*	2
Radiation type	Mo *K*α
μ (mm^−1^)	0.13
Crystal size (mm)	0.44 × 0.22 × 0.08

Data collection	
Diffractometer	Bruker–Nonius Roper CCD camera on κ-goniostat
Absorption correction	Multi-scan (*SADABS*; Sheldrick, 2007 ▸)
*T*_min_, *T*_max_	0.655, 0.746
No. of measured, independent and observed [*I* > 2σ(*I*)] reflections	58243, 10823, 6765
*R*_int_	0.085
(sin θ/λ)_max_ (Å^−1^)	0.649

Refinement	
*R*[*F* ^2^ > 2σ(*F* ^2^)], *wR*(*F* ^2^), *S*	0.077, 0.214, 1.02
No. of reflections	10823
No. of parameters	716
No. of restraints	28
Δρ_max_, Δρ_min_ (e Å^−3^)	1.75, −0.66

Computer programs: *DENZO* (Otwinowski & Minor, 1997 ▸) and *COLLECT* (Hooft, 1998 ▸), *SHELXS97* (Sheldrick, 2008 ▸), *SHELXL2014/7* (Sheldrick, 2015 ▸), *ORTEP-3 for Windows* (Farrugia, 2012 ▸), *QMol* (Gans & Shalloway, 2001 ▸), *DIAMOND* (Brandenburg, 2006 ▸) and *publCIF* (Westrip, 2010 ▸).

## supplementary crystallographic information

### Crystal data

**Table:**

2C_17_H_17_F_6_N_2_O^+^·2C_8_H_7_O_3_^−^·H_2_O	*Z* = 2
*M**_r_* = 1078.95	*F*(000) = 1116
Triclinic, *P*1	*D*_x_ = 1.507 Mg m^−^^3^
*a* = 9.5317 (2) Å	Mo *K*α radiation, λ = 0.71073 Å
*b* = 15.8217 (5) Å	Cell parameters from 36255 reflections
*c* = 16.2980 (5) Å	θ = 2.9–27.5°
α = 85.926 (2)°	µ = 0.13 mm^−^^1^
β = 77.418 (2)°	*T* = 120 K
γ = 83.003 (2)°	Slab, colourless
*V* = 2378.46 (12) Å^3^	0.44 × 0.22 × 0.08 mm

### Data collection

**Table:**

Bruker–Nonius Roper CCD camera on κ-goniostat diffractometer	10823 independent reflections
Radiation source: Bruker–Nonius FR591 rotating anode	6765 reflections with *I* > 2σ(*I*)
Graphite monochromator	*R*_int_ = 0.085
Detector resolution: 9.091 pixels mm^-1^	θ_max_ = 27.5°, θ_min_ = 2.9°
φ & ω scans	*h* = −12→12
Absorption correction: multi-scan (SADABS; Sheldrick, 2007)	*k* = −20→20
*T*_min_ = 0.655, *T*_max_ = 0.746	*l* = −21→21
58243 measured reflections	

### Refinement

**Table:**

Refinement on *F*^2^	28 restraints
Least-squares matrix: full	Hydrogen site location: mixed
*R*[*F*^2^ > 2σ(*F*^2^)] = 0.077	*w* = 1/[σ^2^(*F*_o_^2^) + (0.0944*P*)^2^ + 3.7317*P*] where *P* = (*F*_o_^2^ + 2*F*_c_^2^)/3
*wR*(*F*^2^) = 0.214	(Δ/σ)_max_ = 0.001
*S* = 1.02	Δρ_max_ = 1.75 e Å^−^^3^
10823 reflections	Δρ_min_ = −0.66 e Å^−^^3^
716 parameters	

### Special details

**Table:**

Geometry. All esds (except the esd in the dihedral angle between two l.s. planes) are estimated using the full covariance matrix. The cell esds are taken into account individually in the estimation of esds in distances, angles and torsion angles; correlations between esds in cell parameters are only used when they are defined by crystal symmetry. An approximate (isotropic) treatment of cell esds is used for estimating esds involving l.s. planes.

### Fractional atomic coordinates and isotropic or equivalent isotropic displacement parameters (Å^2^)

**Table:**

	*x*	*y*	*z*	*U*_iso_*/*U*_eq_	Occ. (<1)
F1	0.5340 (2)	0.43419 (13)	0.20495 (13)	0.0349 (5)	
F2	0.6128 (2)	0.42025 (13)	0.07246 (14)	0.0405 (5)	
F3	0.7404 (2)	0.36447 (13)	0.15967 (17)	0.0471 (6)	
F4	1.1534 (2)	0.43806 (13)	0.05811 (12)	0.0333 (5)	
F5	1.1147 (2)	0.44735 (13)	0.19238 (12)	0.0330 (5)	
F6	1.3016 (2)	0.49571 (14)	0.11350 (15)	0.0428 (6)	
O1	0.4545 (2)	0.74208 (14)	0.14151 (15)	0.0252 (5)	
H1O	0.411 (4)	0.7905 (12)	0.153 (3)	0.038*	
N1	0.8612 (3)	0.50764 (16)	0.13007 (16)	0.0213 (6)	
N2	0.5620 (3)	0.86797 (17)	0.00056 (17)	0.0227 (6)	
H1N	0.613 (3)	0.897 (2)	0.026 (2)	0.027*	
H2N	0.4691 (14)	0.877 (2)	0.024 (2)	0.027*	
C1	0.7209 (3)	0.5153 (2)	0.1360 (2)	0.0226 (7)	
C2	0.6315 (3)	0.5923 (2)	0.1303 (2)	0.0226 (7)	
H2	0.5300	0.5924	0.1366	0.027*	
C3	0.6935 (3)	0.66691 (19)	0.11559 (19)	0.0199 (6)	
C4	0.8463 (3)	0.6635 (2)	0.10880 (19)	0.0200 (6)	
C5	0.9235 (3)	0.7364 (2)	0.0943 (2)	0.0246 (7)	
H5	0.8731	0.7914	0.0882	0.030*	
C6	1.0696 (3)	0.7282 (2)	0.0889 (2)	0.0284 (8)	
H6	1.1195	0.7775	0.0791	0.034*	
C7	1.1470 (3)	0.6478 (2)	0.0976 (2)	0.0270 (7)	
H7	1.2484	0.6434	0.0939	0.032*	
C8	1.0773 (3)	0.5755 (2)	0.1114 (2)	0.0234 (7)	
C9	0.9251 (3)	0.5821 (2)	0.11715 (19)	0.0199 (6)	
C10	0.6531 (3)	0.4329 (2)	0.1444 (2)	0.0259 (7)	
C11	1.1601 (3)	0.4896 (2)	0.1193 (2)	0.0270 (7)	
C12	0.6026 (3)	0.75057 (19)	0.10484 (19)	0.0202 (6)	
H12	0.6359	0.7956	0.1338	0.024*	
C13	0.6213 (3)	0.7760 (2)	0.0100 (2)	0.0224 (7)	
H13	0.7272	0.7708	−0.0156	0.027*	
C14	0.5876 (4)	0.9001 (2)	−0.0895 (2)	0.0298 (8)	
H14A	0.6929	0.8978	−0.1129	0.036*	
H14B	0.5453	0.9602	−0.0930	0.036*	
C15	0.5196 (4)	0.8464 (2)	−0.1410 (2)	0.0334 (8)	
H15A	0.5437	0.8657	−0.2010	0.040*	
H15B	0.4131	0.8543	−0.1219	0.040*	
C16	0.5736 (4)	0.7526 (2)	−0.1317 (2)	0.0319 (8)	
H16A	0.6780	0.7435	−0.1576	0.038*	
H16B	0.5218	0.7186	−0.1618	0.038*	
C17	0.5493 (4)	0.7224 (2)	−0.0388 (2)	0.0244 (7)	
H17A	0.4443	0.7264	−0.0143	0.029*	
H17B	0.5894	0.6619	−0.0341	0.029*	
C27	−0.1849 (4)	0.6033 (2)	0.6130 (2)	0.0283 (7)	0.775 (3)
F7	−0.3173 (3)	0.60957 (19)	0.6595 (3)	0.0600 (10)	0.775 (3)
F8	−0.1845 (6)	0.6196 (3)	0.5363 (2)	0.0861 (14)	0.775 (3)
F9	−0.1283 (4)	0.66770 (18)	0.6386 (3)	0.0642 (11)	0.775 (3)
C27'	−0.1849 (4)	0.6033 (2)	0.6130 (2)	0.0283 (7)	0.225 (3)
F7'	−0.2815 (11)	0.5956 (7)	0.5611 (10)	0.0600 (10)	0.225 (3)
F8'	−0.237 (3)	0.6462 (11)	0.6684 (8)	0.0861 (14)	0.225 (3)
F9'	−0.1069 (12)	0.6560 (7)	0.5570 (12)	0.0642 (11)	0.225 (3)
F10	0.3229 (2)	0.55590 (14)	0.53309 (14)	0.0421 (5)	
F11	0.4729 (2)	0.51710 (15)	0.61302 (17)	0.0474 (6)	
F12	0.2720 (2)	0.59052 (14)	0.66265 (15)	0.0430 (6)	
O2	−0.3279 (2)	0.30526 (14)	0.69410 (15)	0.0257 (5)	
H2O	−0.367 (4)	0.298 (3)	0.7449 (9)	0.039*	
N3	0.0360 (3)	0.52001 (16)	0.62328 (17)	0.0226 (6)	
N4	−0.1684 (3)	0.14436 (18)	0.63121 (19)	0.0284 (6)	
H3N	−0.2635 (12)	0.149 (2)	0.643 (2)	0.034*	
H4N	−0.145 (4)	0.125 (2)	0.6788 (14)	0.034*	
C18	−0.1027 (3)	0.5187 (2)	0.6291 (2)	0.0225 (7)	
C19	−0.1772 (3)	0.4453 (2)	0.6498 (2)	0.0233 (7)	
H19	−0.2781	0.4488	0.6521	0.028*	
C20	−0.1013 (3)	0.3697 (2)	0.66638 (19)	0.0211 (6)	
C21	0.0496 (3)	0.3677 (2)	0.66514 (19)	0.0214 (6)	
C22	0.1391 (3)	0.2939 (2)	0.6849 (2)	0.0250 (7)	
H22	0.0981	0.2421	0.7024	0.030*	
C23	0.2832 (4)	0.2969 (2)	0.6789 (2)	0.0300 (8)	
H23	0.3417	0.2468	0.6916	0.036*	
C24	0.3472 (4)	0.3729 (2)	0.6542 (2)	0.0294 (8)	
H24	0.4482	0.3733	0.6496	0.035*	
C25	0.2650 (3)	0.4457 (2)	0.6367 (2)	0.0250 (7)	
C26	0.1131 (3)	0.4453 (2)	0.64167 (19)	0.0221 (7)	
C28	0.3315 (4)	0.5276 (2)	0.6113 (2)	0.0323 (8)	
C29	−0.1749 (3)	0.2881 (2)	0.6832 (2)	0.0219 (7)	
H29	−0.1506	0.2569	0.7348	0.026*	
C30	−0.1209 (4)	0.2313 (2)	0.6072 (2)	0.0240 (7)	
H30	−0.0128	0.2254	0.5944	0.029*	
C31	−0.1103 (4)	0.0833 (2)	0.5618 (2)	0.0334 (8)	
H31A	−0.1445	0.0269	0.5795	0.040*	
H31B	−0.0033	0.0762	0.5505	0.040*	
C32	−0.1608 (4)	0.1170 (2)	0.4828 (2)	0.0365 (9)	
H32A	−0.2675	0.1202	0.4931	0.044*	
H32B	−0.1198	0.0776	0.4370	0.044*	
C33	−0.1134 (4)	0.2050 (2)	0.4565 (2)	0.0361 (8)	
H33A	−0.1507	0.2273	0.4061	0.043*	
H33B	−0.0065	0.2010	0.4417	0.043*	
C34	−0.1709 (4)	0.2659 (2)	0.5282 (2)	0.0290 (7)	
H34A	−0.1360	0.3223	0.5112	0.035*	
H34B	−0.2779	0.2736	0.5396	0.035*	
O3	0.2669 (2)	−0.08127 (14)	0.05126 (15)	0.0292 (5)	
O4	0.2997 (2)	−0.11430 (15)	0.18177 (15)	0.0288 (5)	
O5	0.0214 (2)	−0.09990 (15)	0.24340 (17)	0.0325 (6)	
H5O	−0.0664 (17)	−0.086 (3)	0.266 (3)	0.049*	
C35	0.2226 (3)	−0.08536 (19)	0.1300 (2)	0.0243 (7)	
C36	0.0645 (3)	−0.0537 (2)	0.1657 (2)	0.0263 (7)	
H36	0.0043	−0.0663	0.1259	0.032*	
C37	0.0458 (3)	0.0421 (2)	0.1772 (2)	0.0252 (7)	
C38	−0.0215 (4)	0.0980 (2)	0.1242 (2)	0.0341 (8)	
H38	−0.0560	0.0764	0.0803	0.041*	
C39	−0.0386 (4)	0.1858 (2)	0.1353 (3)	0.0428 (10)	
H39	−0.0853	0.2236	0.0992	0.051*	
C40	0.0119 (4)	0.2175 (2)	0.1985 (3)	0.0445 (10)	
H40	−0.0003	0.2771	0.2062	0.053*	
C41	0.0805 (4)	0.1622 (2)	0.2506 (2)	0.0397 (9)	
H41	0.1165	0.1841	0.2938	0.048*	
C42	0.0972 (4)	0.0748 (2)	0.2405 (2)	0.0316 (8)	
H42	0.1439	0.0374	0.2769	0.038*	
O6	0.7671 (3)	−0.0687 (2)	0.3359 (2)	0.0662 (10)	
O7	0.6636 (4)	−0.0738 (2)	0.2270 (2)	0.0719 (10)	
O8	0.3996 (4)	−0.0457 (2)	0.3191 (2)	0.0648 (9)	
H8O	0.457 (6)	−0.079 (3)	0.284 (3)	0.097*	
C43	0.6642 (5)	−0.0604 (3)	0.3013 (3)	0.0423 (10)	
C44	0.5166 (4)	−0.0306 (3)	0.3535 (3)	0.0456 (10)	
H44	0.5071	−0.0645	0.4082	0.055*	
C45	0.5065 (4)	0.0622 (3)	0.3748 (3)	0.0425 (10)	
C46	0.6000 (5)	0.1182 (3)	0.3294 (3)	0.0514 (11)	
H46	0.6741	0.0987	0.2835	0.062*	
C47	0.5851 (7)	0.2031 (3)	0.3511 (4)	0.0690 (17)	
H47	0.6472	0.2416	0.3190	0.083*	
C48	0.4812 (8)	0.2309 (4)	0.4184 (5)	0.084 (2)	
H48	0.4731	0.2883	0.4341	0.100*	
C49	0.3880 (6)	0.1760 (4)	0.4637 (4)	0.0741 (17)	
H49	0.3150	0.1959	0.5099	0.089*	
C50	0.4008 (5)	0.0919 (3)	0.4419 (3)	0.0563 (12)	
H50	0.3363	0.0543	0.4734	0.068*	
O1W	0.7552 (3)	−0.05535 (16)	0.06281 (17)	0.0358 (6)	
H1W	0.724 (5)	−0.046 (3)	0.1140 (10)	0.054*	
H2W	0.765 (5)	−0.0084 (15)	0.036 (3)	0.054*	

### Atomic displacement parameters (Å^2^)

**Table:**

	*U*^11^	*U*^22^	*U*^33^	*U*^12^	*U*^13^	*U*^23^
F1	0.0299 (11)	0.0308 (11)	0.0418 (12)	−0.0101 (9)	0.0000 (9)	0.0023 (9)
F2	0.0481 (13)	0.0358 (12)	0.0431 (13)	−0.0184 (10)	−0.0114 (10)	−0.0089 (10)
F3	0.0349 (12)	0.0177 (10)	0.0901 (19)	−0.0017 (9)	−0.0193 (12)	0.0064 (11)
F4	0.0330 (11)	0.0343 (11)	0.0306 (11)	0.0092 (9)	−0.0070 (8)	−0.0091 (9)
F5	0.0359 (11)	0.0346 (11)	0.0286 (11)	0.0022 (9)	−0.0117 (9)	0.0023 (9)
F6	0.0195 (10)	0.0433 (13)	0.0660 (16)	0.0022 (9)	−0.0144 (10)	0.0020 (11)
O1	0.0188 (11)	0.0223 (12)	0.0300 (13)	0.0021 (9)	0.0014 (9)	0.0013 (10)
N1	0.0213 (13)	0.0202 (14)	0.0229 (14)	−0.0019 (10)	−0.0061 (10)	−0.0008 (11)
N2	0.0250 (14)	0.0178 (14)	0.0267 (15)	−0.0040 (11)	−0.0076 (11)	−0.0001 (11)
C1	0.0222 (16)	0.0225 (17)	0.0230 (16)	−0.0036 (13)	−0.0039 (12)	−0.0021 (13)
C2	0.0197 (15)	0.0212 (16)	0.0266 (17)	0.0002 (12)	−0.0050 (13)	−0.0022 (13)
C3	0.0186 (15)	0.0209 (16)	0.0202 (16)	−0.0012 (12)	−0.0038 (12)	−0.0033 (12)
C4	0.0187 (15)	0.0214 (16)	0.0194 (15)	−0.0019 (12)	−0.0023 (12)	−0.0029 (12)
C5	0.0277 (17)	0.0200 (16)	0.0262 (17)	−0.0045 (13)	−0.0045 (13)	−0.0023 (13)
C6	0.0245 (17)	0.0313 (19)	0.0319 (19)	−0.0118 (14)	−0.0049 (14)	−0.0066 (15)
C7	0.0190 (15)	0.035 (2)	0.0278 (18)	−0.0056 (14)	−0.0038 (13)	−0.0053 (15)
C8	0.0218 (16)	0.0284 (18)	0.0211 (16)	−0.0027 (13)	−0.0065 (12)	−0.0022 (13)
C9	0.0199 (15)	0.0233 (16)	0.0161 (15)	−0.0016 (12)	−0.0031 (12)	−0.0029 (12)
C10	0.0230 (16)	0.0212 (17)	0.0338 (19)	−0.0023 (13)	−0.0070 (14)	−0.0001 (14)
C11	0.0208 (16)	0.0335 (19)	0.0271 (18)	−0.0010 (14)	−0.0068 (13)	−0.0019 (15)
C12	0.0175 (14)	0.0207 (16)	0.0219 (16)	−0.0033 (12)	−0.0027 (12)	−0.0005 (12)
C13	0.0199 (15)	0.0209 (16)	0.0252 (17)	−0.0034 (12)	−0.0017 (12)	−0.0005 (13)
C14	0.041 (2)	0.0250 (18)	0.0252 (18)	−0.0101 (15)	−0.0086 (15)	0.0036 (14)
C15	0.047 (2)	0.033 (2)	0.0247 (18)	−0.0154 (17)	−0.0143 (16)	0.0057 (15)
C16	0.041 (2)	0.0301 (19)	0.0276 (19)	−0.0142 (16)	−0.0084 (15)	−0.0028 (15)
C17	0.0282 (17)	0.0196 (16)	0.0269 (17)	−0.0062 (13)	−0.0072 (13)	−0.0015 (13)
C27	0.0230 (17)	0.0257 (18)	0.036 (2)	−0.0056 (14)	−0.0062 (14)	0.0033 (15)
F7	0.0242 (14)	0.0260 (15)	0.114 (3)	0.0047 (12)	0.0081 (16)	0.0197 (17)
F8	0.151 (3)	0.062 (2)	0.0317 (17)	0.062 (2)	−0.0295 (19)	−0.0083 (15)
F9	0.0495 (19)	0.0212 (15)	0.131 (3)	−0.0028 (13)	−0.038 (2)	−0.0085 (18)
C27'	0.0230 (17)	0.0257 (18)	0.036 (2)	−0.0056 (14)	−0.0062 (14)	0.0033 (15)
F7'	0.0242 (14)	0.0260 (15)	0.114 (3)	0.0047 (12)	0.0081 (16)	0.0197 (17)
F8'	0.151 (3)	0.062 (2)	0.0317 (17)	0.062 (2)	−0.0295 (19)	−0.0083 (15)
F9'	0.0495 (19)	0.0212 (15)	0.131 (3)	−0.0028 (13)	−0.038 (2)	−0.0085 (18)
F10	0.0356 (12)	0.0451 (13)	0.0443 (14)	−0.0130 (10)	−0.0058 (10)	0.0135 (10)
F11	0.0226 (11)	0.0484 (14)	0.0739 (17)	−0.0110 (10)	−0.0136 (10)	0.0023 (12)
F12	0.0383 (12)	0.0349 (12)	0.0593 (15)	−0.0106 (10)	−0.0116 (11)	−0.0113 (11)
O2	0.0208 (11)	0.0266 (12)	0.0277 (13)	−0.0041 (9)	−0.0007 (9)	0.0018 (10)
N3	0.0234 (14)	0.0209 (14)	0.0238 (14)	−0.0029 (11)	−0.0055 (11)	−0.0011 (11)
N4	0.0378 (16)	0.0196 (14)	0.0285 (16)	−0.0053 (12)	−0.0080 (13)	0.0004 (12)
C18	0.0217 (16)	0.0216 (16)	0.0240 (17)	−0.0039 (12)	−0.0040 (12)	−0.0003 (13)
C19	0.0189 (15)	0.0240 (17)	0.0271 (17)	−0.0034 (12)	−0.0045 (13)	−0.0012 (13)
C20	0.0222 (15)	0.0240 (17)	0.0163 (15)	−0.0026 (13)	−0.0020 (12)	−0.0026 (12)
C21	0.0240 (16)	0.0230 (16)	0.0182 (15)	−0.0026 (13)	−0.0066 (12)	−0.0018 (12)
C22	0.0285 (17)	0.0237 (17)	0.0240 (17)	−0.0023 (13)	−0.0085 (13)	−0.0002 (13)
C23	0.0299 (18)	0.0295 (19)	0.0312 (19)	0.0049 (14)	−0.0117 (14)	−0.0018 (15)
C24	0.0211 (16)	0.038 (2)	0.0306 (19)	−0.0016 (14)	−0.0094 (14)	−0.0044 (15)
C25	0.0237 (16)	0.0316 (19)	0.0212 (17)	−0.0051 (14)	−0.0071 (13)	−0.0004 (14)
C26	0.0249 (16)	0.0240 (17)	0.0173 (15)	−0.0012 (13)	−0.0050 (12)	−0.0015 (12)
C28	0.0213 (17)	0.034 (2)	0.042 (2)	−0.0063 (14)	−0.0068 (15)	−0.0006 (17)
C29	0.0231 (16)	0.0200 (16)	0.0213 (16)	−0.0032 (12)	−0.0030 (12)	0.0036 (12)
C30	0.0283 (17)	0.0189 (16)	0.0249 (17)	−0.0047 (13)	−0.0049 (13)	0.0006 (13)
C31	0.042 (2)	0.0251 (18)	0.0308 (19)	0.0014 (15)	−0.0039 (15)	−0.0075 (15)
C32	0.047 (2)	0.030 (2)	0.034 (2)	0.0004 (16)	−0.0114 (17)	−0.0107 (16)
C33	0.044 (2)	0.036 (2)	0.0285 (19)	−0.0013 (16)	−0.0079 (16)	−0.0050 (16)
C34	0.0361 (19)	0.0257 (18)	0.0255 (18)	−0.0032 (14)	−0.0077 (14)	−0.0001 (14)
O3	0.0315 (13)	0.0247 (12)	0.0308 (14)	−0.0021 (10)	−0.0069 (10)	0.0028 (10)
O4	0.0257 (12)	0.0280 (13)	0.0330 (13)	0.0040 (10)	−0.0095 (10)	−0.0049 (10)
O5	0.0215 (12)	0.0269 (13)	0.0456 (16)	−0.0025 (10)	−0.0025 (11)	0.0091 (11)
C35	0.0263 (16)	0.0141 (15)	0.0335 (19)	−0.0024 (12)	−0.0083 (14)	−0.0022 (13)
C36	0.0248 (16)	0.0188 (16)	0.038 (2)	−0.0027 (13)	−0.0124 (14)	0.0023 (14)
C37	0.0234 (16)	0.0196 (16)	0.0301 (18)	0.0002 (13)	−0.0018 (13)	0.0003 (13)
C38	0.0279 (18)	0.032 (2)	0.041 (2)	0.0010 (15)	−0.0078 (15)	0.0016 (16)
C39	0.040 (2)	0.027 (2)	0.053 (3)	0.0091 (16)	−0.0018 (19)	0.0087 (18)
C40	0.049 (2)	0.0221 (19)	0.052 (3)	−0.0011 (17)	0.012 (2)	−0.0086 (18)
C41	0.054 (2)	0.033 (2)	0.028 (2)	−0.0122 (18)	0.0069 (17)	−0.0067 (16)
C42	0.0360 (19)	0.0283 (19)	0.0287 (19)	−0.0069 (15)	−0.0022 (15)	0.0026 (15)
O6	0.0442 (18)	0.078 (2)	0.074 (2)	0.0105 (17)	−0.0107 (17)	−0.0176 (19)
O7	0.097 (3)	0.065 (2)	0.044 (2)	−0.008 (2)	0.0053 (18)	−0.0053 (17)
O8	0.051 (2)	0.077 (3)	0.071 (2)	−0.0123 (17)	−0.0144 (17)	−0.0224 (19)
C43	0.047 (2)	0.040 (2)	0.039 (2)	−0.0109 (18)	0.0011 (19)	−0.0085 (18)
C44	0.032 (2)	0.040 (2)	0.067 (3)	−0.0051 (17)	−0.0116 (19)	−0.007 (2)
C45	0.042 (2)	0.038 (2)	0.053 (3)	0.0002 (18)	−0.0236 (19)	−0.0052 (19)
C46	0.064 (3)	0.045 (3)	0.057 (3)	−0.015 (2)	−0.038 (2)	0.006 (2)
C47	0.102 (4)	0.032 (2)	0.097 (4)	−0.018 (3)	−0.075 (4)	0.014 (3)
C48	0.099 (5)	0.045 (3)	0.128 (6)	0.024 (3)	−0.082 (5)	−0.025 (4)
C49	0.068 (3)	0.067 (4)	0.097 (5)	0.028 (3)	−0.051 (3)	−0.033 (3)
C50	0.052 (3)	0.057 (3)	0.063 (3)	0.008 (2)	−0.023 (2)	−0.012 (2)
O1W	0.0501 (16)	0.0289 (14)	0.0337 (14)	−0.0130 (12)	−0.0174 (13)	0.0048 (11)

### Geometric parameters (Å, º)

**Table:**

F1—C10	1.332 (4)	C19—H19	0.9500
F2—C10	1.345 (4)	C20—C21	1.430 (4)
F3—C10	1.326 (4)	C20—C29	1.525 (4)
F4—C11	1.348 (4)	C21—C22	1.420 (5)
F5—C11	1.336 (4)	C21—C26	1.427 (4)
F6—C11	1.347 (4)	C22—C23	1.362 (5)
O1—C12	1.426 (4)	C22—H22	0.9500
O1—H1O	0.839 (10)	C23—C24	1.407 (5)
N1—C1	1.311 (4)	C23—H23	0.9500
N1—C9	1.373 (4)	C24—C25	1.362 (5)
N2—C14	1.497 (4)	C24—H24	0.9500
N2—C13	1.505 (4)	C25—C26	1.433 (4)
N2—H1N	0.881 (10)	C25—C28	1.503 (5)
N2—H2N	0.883 (10)	C29—C30	1.541 (4)
C1—C2	1.409 (4)	C29—H29	1.0000
C1—C10	1.510 (4)	C30—C34	1.513 (5)
C2—C3	1.367 (4)	C30—H30	1.0000
C2—H2	0.9500	C31—C32	1.511 (5)
C3—C4	1.431 (4)	C31—H31A	0.9900
C3—C12	1.513 (4)	C31—H31B	0.9900
C4—C5	1.422 (4)	C32—C33	1.519 (5)
C4—C9	1.422 (4)	C32—H32A	0.9900
C5—C6	1.367 (5)	C32—H32B	0.9900
C5—H5	0.9500	C33—C34	1.533 (5)
C6—C7	1.405 (5)	C33—H33A	0.9900
C6—H6	0.9500	C33—H33B	0.9900
C7—C8	1.372 (5)	C34—H34A	0.9900
C7—H7	0.9500	C34—H34B	0.9900
C8—C9	1.425 (4)	O3—C35	1.260 (4)
C8—C11	1.499 (5)	O4—C35	1.263 (4)
C12—C13	1.547 (4)	O5—C36	1.422 (4)
C12—H12	1.0000	O5—H5O	0.845 (10)
C13—C17	1.516 (4)	C35—C36	1.528 (5)
C13—H13	1.0000	C36—C37	1.524 (4)
C14—C15	1.523 (5)	C36—H36	1.0000
C14—H14A	0.9900	C37—C42	1.389 (5)
C14—H14B	0.9900	C37—C38	1.391 (5)
C15—C16	1.519 (5)	C38—C39	1.399 (5)
C15—H15A	0.9900	C38—H38	0.9500
C15—H15B	0.9900	C39—C40	1.375 (6)
C16—C17	1.532 (5)	C39—H39	0.9500
C16—H16A	0.9900	C40—C41	1.384 (6)
C16—H16B	0.9900	C40—H40	0.9500
C17—H17A	0.9900	C41—C42	1.389 (5)
C17—H17B	0.9900	C41—H41	0.9500
C27—F8	1.259 (5)	C42—H42	0.9500
C27—F7	1.319 (4)	O6—C43	1.223 (5)
C27—F9	1.340 (5)	O7—C43	1.246 (5)
C27—C18	1.503 (5)	O8—C44	1.404 (5)
C27'—F8'	1.153 (13)	O8—H8O	0.861 (10)
C27'—F7'	1.399 (15)	C43—C44	1.517 (6)
C27'—F9'	1.352 (14)	C44—C45	1.519 (6)
C27'—C18	1.503 (5)	C44—H44	1.0000
F10—C28	1.337 (4)	C45—C50	1.382 (6)
F11—C28	1.343 (4)	C45—C46	1.394 (6)
F12—C28	1.337 (4)	C46—C47	1.395 (7)
O2—C29	1.424 (4)	C46—H46	0.9500
O2—H2O	0.835 (10)	C47—C48	1.367 (9)
N3—C18	1.308 (4)	C47—H47	0.9500
N3—C26	1.364 (4)	C48—C49	1.377 (9)
N4—C30	1.500 (4)	C48—H48	0.9500
N4—C31	1.507 (4)	C49—C50	1.387 (7)
N4—H3N	0.880 (10)	C49—H49	0.9500
N4—H4N	0.877 (10)	C50—H50	0.9500
C18—C19	1.416 (4)	O1W—H1W	0.840 (10)
C19—C20	1.362 (5)	O1W—H2W	0.841 (10)
			
C12—O1—H1O	109 (3)	C26—C21—C20	117.0 (3)
C1—N1—C9	116.1 (3)	C23—C22—C21	120.5 (3)
C14—N2—C13	112.2 (3)	C23—C22—H22	119.8
C14—N2—H1N	107 (2)	C21—C22—H22	119.8
C13—N2—H1N	105 (2)	C22—C23—C24	121.3 (3)
C14—N2—H2N	110 (2)	C22—C23—H23	119.3
C13—N2—H2N	113 (2)	C24—C23—H23	119.3
H1N—N2—H2N	110 (3)	C25—C24—C23	120.3 (3)
N1—C1—C2	125.9 (3)	C25—C24—H24	119.8
N1—C1—C10	115.8 (3)	C23—C24—H24	119.8
C2—C1—C10	118.2 (3)	C24—C25—C26	120.3 (3)
C3—C2—C1	118.7 (3)	C24—C25—C28	120.9 (3)
C3—C2—H2	120.6	C26—C25—C28	118.8 (3)
C1—C2—H2	120.6	N3—C26—C21	123.1 (3)
C2—C3—C4	118.4 (3)	N3—C26—C25	117.9 (3)
C2—C3—C12	120.4 (3)	C21—C26—C25	119.1 (3)
C4—C3—C12	121.1 (3)	F10—C28—F12	107.4 (3)
C5—C4—C9	118.3 (3)	F10—C28—F11	106.3 (3)
C5—C4—C3	123.9 (3)	F12—C28—F11	106.0 (3)
C9—C4—C3	117.8 (3)	F10—C28—C25	112.8 (3)
C6—C5—C4	120.5 (3)	F12—C28—C25	113.0 (3)
C6—C5—H5	119.7	F11—C28—C25	110.8 (3)
C4—C5—H5	119.7	O2—C29—C20	111.6 (3)
C5—C6—C7	121.0 (3)	O2—C29—C30	107.5 (3)
C5—C6—H6	119.5	C20—C29—C30	109.1 (2)
C7—C6—H6	119.5	O2—C29—H29	109.5
C8—C7—C6	120.6 (3)	C20—C29—H29	109.5
C8—C7—H7	119.7	C30—C29—H29	109.5
C6—C7—H7	119.7	N4—C30—C34	109.7 (3)
C7—C8—C9	119.7 (3)	N4—C30—C29	108.8 (3)
C7—C8—C11	120.6 (3)	C34—C30—C29	114.4 (3)
C9—C8—C11	119.6 (3)	N4—C30—H30	107.9
N1—C9—C4	122.9 (3)	C34—C30—H30	107.9
N1—C9—C8	117.2 (3)	C29—C30—H30	107.9
C4—C9—C8	119.8 (3)	N4—C31—C32	109.9 (3)
F3—C10—F1	107.4 (3)	N4—C31—H31A	109.7
F3—C10—F2	106.7 (3)	C32—C31—H31A	109.7
F1—C10—F2	106.5 (3)	N4—C31—H31B	109.7
F3—C10—C1	114.1 (3)	C32—C31—H31B	109.7
F1—C10—C1	112.1 (3)	H31A—C31—H31B	108.2
F2—C10—C1	109.7 (3)	C31—C32—C33	110.4 (3)
F5—C11—F6	106.2 (3)	C31—C32—H32A	109.6
F5—C11—F4	106.5 (3)	C33—C32—H32A	109.6
F6—C11—F4	106.0 (3)	C31—C32—H32B	109.6
F5—C11—C8	113.5 (3)	C33—C32—H32B	109.6
F6—C11—C8	111.4 (3)	H32A—C32—H32B	108.1
F4—C11—C8	112.7 (3)	C32—C33—C34	110.2 (3)
O1—C12—C3	109.7 (2)	C32—C33—H33A	109.6
O1—C12—C13	110.5 (2)	C34—C33—H33A	109.6
C3—C12—C13	109.2 (2)	C32—C33—H33B	109.6
O1—C12—H12	109.1	C34—C33—H33B	109.6
C3—C12—H12	109.1	H33A—C33—H33B	108.1
C13—C12—H12	109.1	C30—C34—C33	110.7 (3)
N2—C13—C17	108.9 (3)	C30—C34—H34A	109.5
N2—C13—C12	108.7 (2)	C33—C34—H34A	109.5
C17—C13—C12	114.8 (3)	C30—C34—H34B	109.5
N2—C13—H13	108.1	C33—C34—H34B	109.5
C17—C13—H13	108.1	H34A—C34—H34B	108.1
C12—C13—H13	108.1	C36—O5—H5O	112 (3)
N2—C14—C15	110.5 (3)	O3—C35—O4	124.5 (3)
N2—C14—H14A	109.6	O3—C35—C36	118.0 (3)
C15—C14—H14A	109.6	O4—C35—C36	117.5 (3)
N2—C14—H14B	109.6	O5—C36—C37	111.6 (3)
C15—C14—H14B	109.6	O5—C36—C35	107.3 (3)
H14A—C14—H14B	108.1	C37—C36—C35	111.0 (3)
C14—C15—C16	111.0 (3)	O5—C36—H36	109.0
C14—C15—H15A	109.4	C37—C36—H36	109.0
C16—C15—H15A	109.4	C35—C36—H36	109.0
C14—C15—H15B	109.4	C42—C37—C38	119.1 (3)
C16—C15—H15B	109.4	C42—C37—C36	120.3 (3)
H15A—C15—H15B	108.0	C38—C37—C36	120.6 (3)
C15—C16—C17	110.8 (3)	C37—C38—C39	120.3 (4)
C15—C16—H16A	109.5	C37—C38—H38	119.9
C17—C16—H16A	109.5	C39—C38—H38	119.9
C15—C16—H16B	109.5	C40—C39—C38	120.2 (4)
C17—C16—H16B	109.5	C40—C39—H39	119.9
H16A—C16—H16B	108.1	C38—C39—H39	119.9
C13—C17—C16	110.9 (3)	C39—C40—C41	119.7 (4)
C13—C17—H17A	109.5	C39—C40—H40	120.2
C16—C17—H17A	109.5	C41—C40—H40	120.2
C13—C17—H17B	109.5	C40—C41—C42	120.6 (4)
C16—C17—H17B	109.5	C40—C41—H41	119.7
H17A—C17—H17B	108.1	C42—C41—H41	119.7
F8—C27—F7	111.8 (4)	C41—C42—C37	120.2 (4)
F8—C27—F9	106.1 (4)	C41—C42—H42	119.9
F7—C27—F9	102.5 (3)	C37—C42—H42	119.9
F8—C27—C18	112.8 (3)	C44—O8—H8O	90 (4)
F7—C27—C18	111.3 (3)	O6—C43—O7	128.3 (4)
F9—C27—C18	111.6 (3)	O6—C43—C44	117.7 (4)
F8'—C27'—F7'	111.8 (12)	O7—C43—C44	114.0 (4)
F8'—C27'—F9'	103.4 (13)	O8—C44—C43	114.6 (4)
F7'—C27'—F9'	93.5 (8)	O8—C44—C45	111.2 (3)
F8'—C27'—C18	119.8 (7)	C43—C44—C45	111.4 (3)
F7'—C27'—C18	111.2 (5)	O8—C44—H44	106.3
F9'—C27'—C18	113.9 (5)	C43—C44—H44	106.3
C29—O2—H2O	110 (3)	C45—C44—H44	106.3
C18—N3—C26	117.0 (3)	C50—C45—C46	118.9 (4)
C30—N4—C31	111.8 (3)	C50—C45—C44	118.7 (4)
C30—N4—H3N	109 (3)	C46—C45—C44	122.4 (4)
C31—N4—H3N	110 (3)	C45—C46—C47	120.1 (5)
C30—N4—H4N	111 (3)	C45—C46—H46	119.9
C31—N4—H4N	112 (3)	C47—C46—H46	119.9
H3N—N4—H4N	103 (4)	C48—C47—C46	120.1 (6)
N3—C18—C19	125.1 (3)	C48—C47—H47	120.0
N3—C18—C27'	115.2 (3)	C46—C47—H47	120.0
C19—C18—C27'	119.7 (3)	C47—C48—C49	120.3 (5)
N3—C18—C27	115.2 (3)	C47—C48—H48	119.9
C19—C18—C27	119.7 (3)	C49—C48—H48	119.9
C20—C19—C18	118.7 (3)	C48—C49—C50	120.1 (6)
C20—C19—H19	120.6	C48—C49—H49	120.0
C18—C19—H19	120.6	C50—C49—H49	120.0
C19—C20—C21	119.0 (3)	C45—C50—C49	120.6 (5)
C19—C20—C29	120.5 (3)	C45—C50—H50	119.7
C21—C20—C29	120.5 (3)	C49—C50—H50	119.7
C22—C21—C26	118.5 (3)	H1W—O1W—H2W	109 (5)
C22—C21—C20	124.4 (3)		
			
C9—N1—C1—C2	0.1 (5)	C19—C20—C21—C22	177.1 (3)
C9—N1—C1—C10	176.0 (3)	C29—C20—C21—C22	−4.8 (5)
N1—C1—C2—C3	1.4 (5)	C19—C20—C21—C26	−3.4 (4)
C10—C1—C2—C3	−174.5 (3)	C29—C20—C21—C26	174.7 (3)
C1—C2—C3—C4	−1.6 (5)	C26—C21—C22—C23	−2.0 (5)
C1—C2—C3—C12	177.1 (3)	C20—C21—C22—C23	177.4 (3)
C2—C3—C4—C5	−179.5 (3)	C21—C22—C23—C24	0.8 (5)
C12—C3—C4—C5	1.9 (5)	C22—C23—C24—C25	0.9 (5)
C2—C3—C4—C9	0.5 (4)	C23—C24—C25—C26	−1.3 (5)
C12—C3—C4—C9	−178.1 (3)	C23—C24—C25—C28	179.2 (3)
C9—C4—C5—C6	−0.3 (5)	C18—N3—C26—C21	0.9 (4)
C3—C4—C5—C6	179.7 (3)	C18—N3—C26—C25	−179.2 (3)
C4—C5—C6—C7	0.0 (5)	C22—C21—C26—N3	−178.6 (3)
C5—C6—C7—C8	0.3 (5)	C20—C21—C26—N3	1.9 (4)
C6—C7—C8—C9	−0.2 (5)	C22—C21—C26—C25	1.6 (4)
C6—C7—C8—C11	179.0 (3)	C20—C21—C26—C25	−177.9 (3)
C1—N1—C9—C4	−1.3 (4)	C24—C25—C26—N3	−179.8 (3)
C1—N1—C9—C8	179.3 (3)	C28—C25—C26—N3	−0.3 (4)
C5—C4—C9—N1	−179.0 (3)	C24—C25—C26—C21	0.1 (5)
C3—C4—C9—N1	1.0 (4)	C28—C25—C26—C21	179.5 (3)
C5—C4—C9—C8	0.4 (4)	C24—C25—C28—F10	116.2 (3)
C3—C4—C9—C8	−179.6 (3)	C26—C25—C28—F10	−63.2 (4)
C7—C8—C9—N1	179.3 (3)	C24—C25—C28—F12	−121.7 (4)
C11—C8—C9—N1	0.1 (4)	C26—C25—C28—F12	58.9 (4)
C7—C8—C9—C4	−0.2 (5)	C24—C25—C28—F11	−2.9 (5)
C11—C8—C9—C4	−179.3 (3)	C26—C25—C28—F11	177.7 (3)
N1—C1—C10—F3	11.5 (4)	C19—C20—C29—O2	−10.6 (4)
C2—C1—C10—F3	−172.2 (3)	C21—C20—C29—O2	171.4 (3)
N1—C1—C10—F1	133.8 (3)	C19—C20—C29—C30	108.1 (3)
C2—C1—C10—F1	−49.9 (4)	C21—C20—C29—C30	−69.9 (4)
N1—C1—C10—F2	−108.1 (3)	C31—N4—C30—C34	58.3 (4)
C2—C1—C10—F2	68.2 (4)	C31—N4—C30—C29	−175.9 (3)
C7—C8—C11—F5	121.6 (3)	O2—C29—C30—N4	−70.7 (3)
C9—C8—C11—F5	−59.2 (4)	C20—C29—C30—N4	168.1 (3)
C7—C8—C11—F6	1.8 (4)	O2—C29—C30—C34	52.4 (3)
C9—C8—C11—F6	−179.1 (3)	C20—C29—C30—C34	−68.9 (3)
C7—C8—C11—F4	−117.2 (3)	C30—N4—C31—C32	−58.7 (4)
C9—C8—C11—F4	62.0 (4)	N4—C31—C32—C33	57.5 (4)
C2—C3—C12—O1	20.9 (4)	C31—C32—C33—C34	−56.9 (4)
C4—C3—C12—O1	−160.5 (3)	N4—C30—C34—C33	−56.9 (4)
C2—C3—C12—C13	−100.4 (3)	C29—C30—C34—C33	−179.4 (3)
C4—C3—C12—C13	78.2 (3)	C32—C33—C34—C30	56.8 (4)
C14—N2—C13—C17	−59.3 (3)	O3—C35—C36—O5	151.9 (3)
C14—N2—C13—C12	175.0 (2)	O4—C35—C36—O5	−27.8 (4)
O1—C12—C13—N2	73.3 (3)	O3—C35—C36—C37	−85.9 (4)
C3—C12—C13—N2	−166.0 (2)	O4—C35—C36—C37	94.4 (3)
O1—C12—C13—C17	−48.9 (3)	O5—C36—C37—C42	48.2 (4)
C3—C12—C13—C17	71.8 (3)	C35—C36—C37—C42	−71.4 (4)
C13—N2—C14—C15	58.0 (4)	O5—C36—C37—C38	−132.4 (3)
N2—C14—C15—C16	−54.9 (4)	C35—C36—C37—C38	108.0 (3)
C14—C15—C16—C17	54.4 (4)	C42—C37—C38—C39	−0.8 (5)
N2—C13—C17—C16	57.8 (3)	C36—C37—C38—C39	179.8 (3)
C12—C13—C17—C16	179.8 (3)	C37—C38—C39—C40	0.4 (6)
C15—C16—C17—C13	−56.4 (4)	C38—C39—C40—C41	0.4 (6)
C26—N3—C18—C19	−2.4 (5)	C39—C40—C41—C42	−0.8 (6)
C26—N3—C18—C27'	176.7 (3)	C40—C41—C42—C37	0.5 (5)
C26—N3—C18—C27	176.7 (3)	C38—C37—C42—C41	0.3 (5)
F8'—C27'—C18—N3	−91.4 (16)	C36—C37—C42—C41	179.8 (3)
F7'—C27'—C18—N3	135.7 (6)	O6—C43—C44—O8	−162.3 (4)
F9'—C27'—C18—N3	31.7 (8)	O7—C43—C44—O8	17.3 (6)
F8'—C27'—C18—C19	87.8 (16)	O6—C43—C44—C45	70.2 (5)
F7'—C27'—C18—C19	−45.1 (7)	O7—C43—C44—C45	−110.2 (4)
F9'—C27'—C18—C19	−149.1 (8)	O8—C44—C45—C50	70.9 (5)
F8—C27—C18—N3	86.7 (5)	C43—C44—C45—C50	−159.8 (4)
F7—C27—C18—N3	−146.6 (4)	O8—C44—C45—C46	−109.3 (4)
F9—C27—C18—N3	−32.7 (5)	C43—C44—C45—C46	20.0 (6)
F8—C27—C18—C19	−94.1 (5)	C50—C45—C46—C47	−0.8 (6)
F7—C27—C18—C19	32.6 (5)	C44—C45—C46—C47	179.4 (4)
F9—C27—C18—C19	146.5 (4)	C45—C46—C47—C48	1.8 (7)
N3—C18—C19—C20	0.9 (5)	C46—C47—C48—C49	−1.9 (8)
C27'—C18—C19—C20	−178.2 (3)	C47—C48—C49—C50	1.0 (8)
C27—C18—C19—C20	−178.2 (3)	C46—C45—C50—C49	0.0 (7)
C18—C19—C20—C21	2.1 (5)	C44—C45—C50—C49	179.8 (4)
C18—C19—C20—C29	−175.9 (3)	C48—C49—C50—C45	−0.1 (7)

### Hydrogen-bond geometry (Å, º)

Cg1–Cg4 are the ring centroids of the (C37–C42), (N1,C1–C4,C9), (N3,C18–C21,C26) and (C21–C26) rings, respectively.

**Table:**

*D*—H···*A*	*D*—H	H···*A*	*D*···*A*	*D*—H···*A*
O8—H8*O*···O7	0.86 (5)	2.00 (6)	2.638 (5)	131 (5)
O1—H1*O*···O4^i^	0.84 (2)	1.76 (3)	2.597 (3)	173 (4)
O2—H2*O*···O1^ii^	0.84 (2)	1.95 (2)	2.779 (3)	169 (5)
N2—H1*N*···O1*W*^i^	0.89 (3)	1.85 (3)	2.725 (4)	167 (3)
N2—H2*N*···O3^i^	0.88 (2)	1.93 (2)	2.788 (4)	165 (3)
N4—H3*N*···O8^iii^	0.88 (2)	2.18 (3)	2.798 (5)	127 (3)
N4—H4*N*···O4^iii^	0.88 (3)	2.43 (3)	3.059 (4)	129 (3)
N4—H4*N*···O5^iii^	0.88 (3)	1.90 (3)	2.727 (4)	156 (3)
O5—H5*O*···O6^iv^	0.85 (3)	1.74 (3)	2.572 (4)	165 (5)
O1*W*—H1*W*···O7	0.84 (2)	1.84 (2)	2.635 (4)	156 (5)
O1*W*—H2*W*···O3^v^	0.84 (3)	1.98 (4)	2.768 (3)	156 (5)
C5—H5···O1*W*^i^	0.95	2.59	3.539 (4)	175
C14—H14*A*···*Cg*1^vi^	0.99	2.66	3.642 (4)	171
C11—F4···*Cg*2^vii^	1.35 (1)	2.93 (1)	4.118 (3)	146 (1)
C11—F5···*Cg*3^viii^	1.34 (1)	3.15 (1)	3.931 (3)	117 (1)
C27—F8···*Cg*4^ii^	1.26 (1)	3.23 (1)	4.474 (3)	170 (1)

Symmetry codes: (i) *x*, *y*+1, *z*; (ii) −*x*, −*y*+1, −*z*+1; (iii) −*x*, −*y*, −*z*+1; (iv) *x*−1, *y*, *z*; (v) −*x*+1, −*y*, −*z*; (vi) −*x*+1, −*y*+1, −*z*; (vii) −*x*+2, −*y*+1, −*z*; (viii) −*x*+1, −*y*+1, −*z*+1.
